# Accuracy of the urine point-of-care circulating cathodic antigen assay for diagnosing *Schistosomiasis mansoni* infection in Brazil: A multicenter study

**DOI:** 10.1590/0037-8682-0238-2022

**Published:** 2023-01-23

**Authors:** Otavio Sarmento Pieri, Fernando Schemelzer Moraes Bezerra, Paulo Marcos Zech Coelho, Martin Johannes Enk, Tereza Cristina Favre, Carlos Graeff-Teixeira, Ricardo Riccio Oliveira, Mitermayer Galvão dos Reis, Lee Senhorinha de Almeida Andrade, Lilian Christina Nóbrega Holsbach Beck, Vivian Favero, Thainá Rodrigues de Souza Fialho, Ricardo José de Paula Souza e Guimarães, Bruna Souza Santos Oliveira, Vanessa Fey Pascoal, Marta Cristhiany Cunha Pinheiro, Ronald Alves dos Santos, Luciano Kalabric Silva, Isadora Cristina de Siqueira, Renata Perotto de Souza, Naftale Katz

**Affiliations:** 1 Fundação Oswaldo Cruz, Instituto Oswaldo Cruz, Laboratório de Educação em Ambiente e Saúde, Rio de Janeiro, RJ, Brasil.; 2 Universidade Federal do Ceará, Departamento de Análises Clínicas e Toxicológicas, Fortaleza, CE, Brasil.; 3 Fundação Oswaldo Cruz, Instituto René Rachou, Belo Horizonte, MG, Brasil.; 4Instituto Evandro Chagas, Laboratório de Parasitoses Intestinais, Esquistossomose e Malacologia, Secção de Parasitologia, Ananindeua, PA, Brasil.; 5 Universidade Federal do Espírito Santo, Centro de Ciências da Saúde, Unidade de Doenças Infecciosas, Vitória, ES, Brasil.; 6 Pontifícia Universidade Católica do Rio Grande do Sul, Laboratório de Parasitologia Biomédica, Porto Alegre, RS, Brasil.; 7 Fundação Oswaldo Cruz, Instituto Gonçalo Moniz, Salvador, BA, Brasil.; 8 Universidade Federal da Bahia, Faculdade de Medicina, Salvador, BA, Brasil.; 9 Yale University, School of Public Health, Department of Epidemiology of Microbial Diseases, New Haven, CT, United States of America.

**Keywords:** Schistosoma mansoni, Point-of-care (POC), Circulating cathodic antigen (CCA), Kato-Katz, Helmintex, Brazil

## Abstract

**Background:**

The World Health Organization recommends a market-ready, urine-based point-of-care diagnostic test for circulating cathodic antigens (CCA) to determine the prevalence of *S. mansoni*. This study evaluated the performance of the URINE CCA (SCHISTO) ECO TESTE® (POC-ECO), which is currently available in Brazil.

**Methods:**

Residents from eight sites with different prevalence estimates provided one urine sample for POC-ECO and one stool sample for Kato-Katz (KK) and Helmintex® (HTX) testing as an egg-detecting reference for infection status.

**Results:**

None of the study sites had significantly higher POC-ECO accuracy than KK.

**Conclusions:**

POC-ECO is not currently recommended in Brazilian schistosomiasis elimination programs.

In Brazil, approximately 1% of the population is infected with *Schistosoma mansoni*, mainly in areas with unsafe water supply and poor sanitation^1^. The Brazilian government has endorsed successive World Health Assembly resolutions and World Health Organization recommendations to eliminate schistosomiasis, including the WHO roadmap 2021-2030^2^. In response to the country's ecoepidemiological characteristics and public health policies, the Ministry of Health (MoH) recommends community-wide active search and treatment of infection carriers at the primary care level in low-risk endemic areas, which account for most sites^3^. Currently, this includes regular stool examinations using the KK diagnostic method^4^, followed by administration of praziquantel for egg-positive cases in all age groups if there is no individual contraindication. This test-and-treat regimen is not appropriate for these situations because routine KK (one sample and two slides) cannot detect infected cases with a low egg burden. Studies using robust egg detection methods as a diagnostic reference have shown that a relatively high proportion of egg-positive cases may be missed by routine KK and thus go untreated^5^ ^,^ ^6^.

The WHO recommends the use of a market-ready, urine-based point-of-care circulating cathodic antigen (POC-CCA) assay to determine the prevalence of *S. mansoni* infection^7^; POC-CCA is considered more sensitive than the routine KK and is easy to handle at a low cost^8^. POC-CCA has been available in Brazil since 2017 under the name URINE CCA (SCHISTO) ECO TESTE® (short: POC-ECO). The test is manufactured by ECO Diagnóstica Ltda. under license from Rapid Medical Diagnostics (RMD), South Africa, and is approved by the Brazilian Health Regulatory Agency (Anvisa) for use as an auxiliary diagnostic tool in clinical practice. However, recent studies from Africa and Brazil suggest that the sensitivity and specificity of POC-CCA are lower in areas of moderate and low endemicity than in areas of high endemicity, and there is increasing evidence that it can lead to false positive results^9^ Therefore, the MoH commissioned a multicenter research study to evaluate the performance of the product in the conditions of the Brazilian Schistosomiasis Control Program (PCE), particularly in low-risk settings^10^. This paper describes the results of this study on the accuracy of POC-ECO at representative sites with different levels of endemicity, following the same core protocol. The study protocol was approved by the Ethics Committee of the Oswaldo Cruz Institute-Fiocruz (CAAE: 82469417.8.0000.5248).

Eight sites were selected for the study because the prevalence was already known based on routine municipality-level KK results between 2008 and 2017 (Figure 1). No treatment interventions were performed at any study site in the 12 months before the study. After obtaining written consent from the health authorities in each municipality, research teams contacted study sites to recruit residents aged two years or older. Individuals with self-reported pregnancy or clinical signs/symptoms of acute illness, severe chronic diseases, and praziquantel treatment within the past 12 months were excluded. Those who agreed to provide free, informed consent were given collection vials and asked to provide ~10 mL of midstream morning urine and ~50 g of stool (one sample each) to be refrigerated until the next day. Samples were then collected by the research teams and transported in cool containers to a local laboratory, where they were immediately processed. Urine samples were tested using POC-ECO according to the manufacturer's instruction leaflet issue 001/2018, which was included in the kit^10^. The outcome was graded by G-scores, as described by Casacuberta-Partal *et al*.^11^. Batch 180907091 was used at all sites except Palmital, where batch 201806011 was used for operational reasons. Stool samples were processed and tested by the KK and Helmintex® (HTX) methods, as described by Graeff-Teixeira *et al*.^9^. Subjects who tested positive for *S. mansoni* eggs by the KK or HTX method and those who showed soil-transmitted helminths by the KK method were referred to a local health facility for treatment, as described by MoH^3^.

**FIGURE 1:**
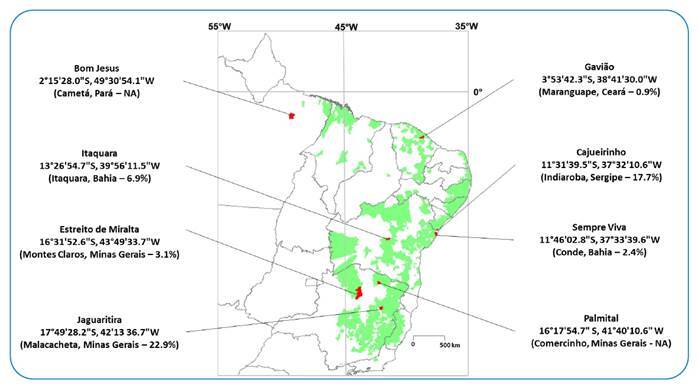
Geographic distribution and DMS coordinates of study sites. Municipalities (in red) and states are indicated in parentheses. The area served by the Brazilian Schistosomiasis Control Program in 2008-2017 is shown in green. The prevalence of *Schistosoma mansoni* infection based on the total number of Kato-Katz exams recorded at the municipality level during this period is shown as percentage (%). Source: http://tabnet.datasus.gov.br/cgi/deftohtm.exe?sinan/pce/cnv/pcebr.def Assessed March 24, 2022). NA, not available.

Research teams aimed at testing 300 participants per site, based on recommendations from Bujang and Adnan^12^ for evaluating the sensitivity and specificity of the diagnostic tests with a statistical power of 80% and a significance level of 5% (α = 0.05). Every effort was made to include as many participants as possible. No randomization procedure was used in the selection of the participants. The following data were transferred from the individual case record sheets at each study site to an Excel spreadsheet (see Supplementary Material 1): Age (years), sex (female/male), POC-ECO results (G1, negative; G2-G3, traces; G4-G10, positive), number of *S. mansoni* eggs per KK slide, number of *S. mansoni* eggs and the amount of stool (grams) tested with HTX. Only data from fully compliant participants were analyzed. Test results (positive/negative) per site are given as absolute numbers and percentages with 95% confidence intervals (CIs). Non-overlapping CIs indicate a significant difference between the percentages compared. For overlapping CIs, the CI was calculated for the difference between the percentages (https://www.statology.org/confidence-interval-difference-in-proportions-calculator/, accessed March 24, 2022), and the difference was considered significant if the resulting CI did not contain zero^13^. POC-ECO positivity was calculated by classifying traces as positive (t+) or negative (t-). KK and HTX positivity was calculated separately and in combination (KK + HTX) (Table 1). Performance parameters of POC-ECO (t+ and t-) and KK were estimated using the MedCalc Diagnostic Test Evaluation Calculator (https://www.medcalc.org/calc/diagnostic_test.php, accessed March 24, 2022), with HTX used as a reference for infection status (see Supplementary Material 2). Figure 2 displays the accuracy of POC-ECO t+, POC-ECO t-, and KK, which was calculated as the overall probability that HTX-negative and positive cases were correctly classified and given by the formula: specificity × prevalence + specificity × (1 − prevalence).

**TABLE 1:** Results from POC-ECO, Helmintex (HTX), and Kato-Katz (KK) from eight sites ranked by KK (one sample, two slides) positivity for *Schistosoma mansoni*. Urine was tested using POC-ECO batch 180907091 (except Palmital, where batch 201806011 was used), with traces scored as positive (t+) or negative (t-). The combined result of HTX and KK (KK+HTX) was used as a reference for infection status. N, the total number of fully compliant subjects; n, number of subjects by outcome (positive/negative); CI, confidence interval. Non-overlapping CIs indicate a significant difference between percentages at an alpha level of 0.05 (5%).

			KK+HTX			POC-ECO t+			POC-ECO t-			Helmintex			Kato-Katz	
Site (N)	Outcome		n	% positives (95% CI)		n	% positives (95% CI)		n	% positives (95% CI)		n	% positives (95% CI)		n	% positives (95% CI)
Sempre Viva (381)	Negative		222			122			216			246			277	
	Positive		159	41.7 (36.7-46.9)		259	68.0 (62.2-73.2)		165	43.3 (37.5-49.1)		135	35.4 (29.9-41.1)		104	27.3 (22.2-32.7)
Jaguaritira (242)	Negative		101			55			103			107			193	
	Positive		141	58.3 (51.8-64.6)		187	77.3 (70.5-83.0)		139	57.4 (50.0-64.5)		135	55.8 (48.2-62.9)		49	20.2 (14.6-26.6)
Cajueirinho (255)	Negative		174			101			156			176			224	
	Positive		81	31.8 (26.1-37.9)		154	60.4 (53.1-67.1)		99	38.8 (31.9-45.9)		79	31.0 (24.5-37.8)		31	12.2 (7.9-17.4)
Itaquara (289)	Negative		238			163			253			243			271	
	Positive		51	17.7 (13.4-22.5)		126	43.6 (36.9-50.3)		36	12.5 (8.4-17.4)		46	15.9 (11.3-21.3)		18	6.2 (3.4-10.1)
Gavião (286)	Negative		263			88			213			264			279	
	Positive		23	8.0 (5.2-11.8)		198	69.2 (62.6-75.1)		73	25.5 (19.8-31.7)		22	7.7 (4.5-11.1)		7	2.4 (0.8-5.3)
Est. Miralta (98)	Negative		96			56			81			96			96	
	Positive		2	2.0 (0.2-7.2)		42	42.9 (31.3-54.4)		17	17.3 (9.4-27.4)		2	2.0 (0.1-7.8)		2	2.0 (0.1-7.8)
Palmital (97)	Negative		93			95			95			93			96	
	Positive		4	4.1 (1.1-10.2)		2	2.1 (0.1-7.9)		2	2.1 (0.1-7.9)		4	4.1 (0.7-11.0)		1	1.0 (0.0-6.2)
Bom Jesus (247)	Negative		246			213			247			246			246	
	Positive		1	0.4 (0.0-2.2)		34	13.8 (9.1-19.3)		0	0 (0.0-1.8)		1	0.4 (0.0-2.5)		1	0.4 (0.0-2.5)

**FIGURE 2:**
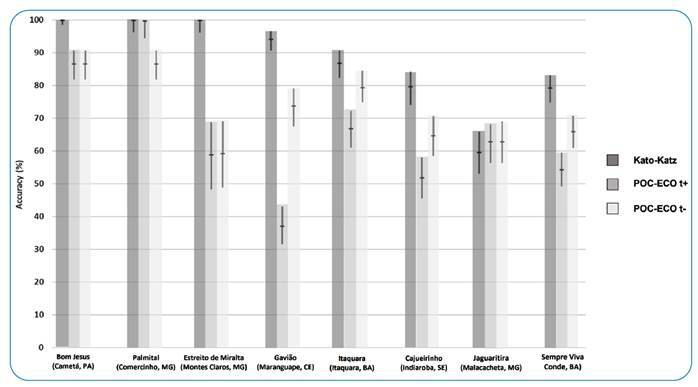
Accuracy of Kato-Katz and POC-ECO considering Helmintex® results as reference. POC-ECO results were calculated considering traces as positive (t+) and negative (t-). The confidence intervals (CIs) of 95% are shown as vertical lines. Non-overlapping CIs indicate significant differences (p < 0.05) in accuracy; overlapping CIs indicate non-significant differences.

Of the 1,895 fully compliant participants, 462 (24.4%) were egg-positive at KK + HTX. The rate of heavy-intensity infections was 0.6%, as there were 12 participants with ≥400 eggs per gram (epg) in feces at KK: Five in Sempre Viva and seven in Jaguaritira. In Bom Jesus, a non-endemic locality, the only egg-positive case was confirmed as allochthonous. POC-ECO t+ yielded significantly higher positivity rates than KK + HTX in all study sites except Palmital. POC-ECO t- yielded significantly higher positivity rates than KK + HTX in Gavião and Estreito de Miralta. POC-ECO t+ positivity rates were significantly higher than POC-ECO t- in all study sites except Palmital. Egg-positivity rates were significantly different between KK and HTX in Jaguaritira, Cajueirinho, and Itaquara (Table 1). The accuracy of POC-ECO t+ was significantly lower than that of KK, except in Palmital and Jaguaritira; that of POC-ECO t- was significantly lower than that of KK, except in Jaguaritira; and that of POC-ECO t+ was significantly lower than that of POC-ECO t- in Gavião, Itaquara, Cajueirinho, and Sempre Viva. At none of the study sites was the accuracy of POC-ECO (either t+ or t-) significantly higher than that of KK (Figure 2).

POC-ECO t+ did not reliably reflect individual infection status (positive or negative) as determined by a robust egg detection reference, which compromised the correct identification of infected and uninfected individuals. Additionally, it failed to reliably estimate the prevalence of infection in low-to-moderate prevalence settings (<50% by KK, according to the WHO^7^). Community-wide POC-ECO t+ positivity in Gavião (69.2%) far exceeded the threshold of 30% using the POC-CCA assay recommended by the WHO^7^ for classifying low-prevalence settings. The POC-ECO t+ positivity rate among fully compliant children aged 6-15 years was 82.9% (29 of 35). This would lead Gavião to a biannual MDA regimen, as it would be classified as a high-prevalence area under the WHO^7^. In Bom Jesus, the only egg-positive case was detected by KK, HTX, and POC-ECO t+. However, the latter yielded 34 positives, resulting in a positivity rate of 13.8% in the community as a whole and 21.7% (13 out of 60) in the 6-15 age group; this POC-ECO t+ result would incorrectly classify Bom Jesus as eligible for MDA according to the WHO^7^. Also, in Estreito de Miralta, only two of the 98 participants were positive for *S. mansoni* eggs, and POC-ECO t+ yielded 42 positive results. In contrast, in Palmital, there were four egg positives among the 97 participants, and POC-ECO t+ yielded only two positives (Supplementary materials 1 and 2). Because similar epidemiological conditions prevailed at Estreito de Miralta and Palmital, the discrepancy in POC-ECO t+ performance between the two sites could be due to the different POC-ECO batches used, as reported by Viana *et al*.^14^. Graeff-Teixeira *et al*.^9^ and Favre *et al.* ^10^ found high levels of trace results with batch 180907091, which may explain the significantly higher positivity of POC-ECO t+ versus POC-ECO t- at all sites except Palmital, where batch 201806011 was used. However, the manufacturer's instructions for use state that POC-ECO t- is not suitable for diagnosis because the presence of two lines in the result window, regardless of the intensity of the test line, should be considered a reactive result. Overall, the sensitivity (true-positive rate) of POC-ECO t+ was over 80% (80.2%; 95% CI:76.1% - 83.9%), while the specificity (true-negative rate) was less than 60% (55.0%; 95% CI:52.4% - 57.6%). The high false positives of POC-ECO t+ found at sites of varying prevalence confirm previous results from a non-endemic area^9^ and reiterate concerns by the WHO^15^ regarding the reliability of current formulations of the test.

The MoH is currently updating the Schistosomiasis Action Plan^9^ in accordance with the WHO targets and sub-targets for the elimination of *S. mansoni* as a public health problem by 2030^2^ and adapting the recommendations of the new WHO guideline^7^ to a country-specific context. After decades of community-based interventions by PCE, endemic areas have achieved a very low overall rate of heavy-intensity infections, as noted in this study. Of the 9.15 million KK tests conducted by the PCE from 2008 to 2017, only 27,149 (0.3%) had more than 400 *S. mansoni* epg (http://tabnet.datasus.gov.br/cgi/deftohtm.exe?sinan/pce/cnv/pcebr.def, accessed March 24, 2022). In this way, the country will most likely succeed in validating schistosomiasis elimination as a public health problem (currently defined as < 1% proportion of heavy-intensity infections^2^) in due time. Since the target moves on to interrupting transmission, verification will depend on developing a highly accurate diagnostic test, which is not currently available^2^. The WHO^7^ proposes a verification framework that involves the use of a high-sensitivity test followed by a high-specificity confirmatory test. If this two-step process was employed in this study, the use of POC-ECO t+ as the first step would reveal 1,002 positive cases, 182 of which would be confirmed by KK; this process would miss 280 (60.6%) of the 462 participants who tested positive at KK + HTX (see Supplementary Material 1). The unsatisfactory performance of POC-ECO in areas with low egg burden has been reported in the manufacturer's package insert 001/2018 edition^10^. In conclusion, POC-ECO is not currently recommended as a diagnostic tool for monitoring and evaluating control measures to eliminate *S. mansoni* infection in Brazil.
