# Supplementary material for: Accuracy of the urine point-of-care circulating cathodic antigen assay for diagnosing Schistosomiasis mansoni infection in Brazil: A multicenter study
Source: Rev Soc Bras Med Trop. 2023 Jan 23;56:e0238-2022. doi: 10.1590/0037-8682-0238-2022 (PMC9870275; doi:10.1590/0037-8682-0238-2022)
Supplement: Supplementary file 1 [file 1678-9849-rsbmt-56-e0238-2022-supp1.pdf]

Site	Age	Gender	G-score	SmKK1	SmKK2	SmHTX	g
Bom Jesus	2	F	G2	0	0	0	30
Bom Jesus	2	F	G3	0	0	0	30
Bom Jesus	2	M	G1	0	0	0	30
Bom Jesus	3	F	G1	0	0	0	30
Bom Jesus	3	M	G3	0	0	0	30
Bom Jesus	3	F	G1	0	0	0	30
Bom Jesus	4	F	G1	0	0	0	30
Bom Jesus	4	M	G1	0	0	0	30
Bom Jesus	4	F	G1	0	0	0	30
Bom Jesus	4	F	G1	0	0	0	30
Bom Jesus	4	F	G1	0	0	0	30
Bom Jesus	4	M	G1	0	0	0	30
Bom Jesus	4	F	G2	0	0	0	30
Bom Jesus	4	M	G1	0	0	0	30
Bom Jesus	4	M	G2	0	0	0	30
Bom Jesus	4	F	G1	0	0	0	30
Bom Jesus	5	M	G1	0	0	0	30
Bom Jesus	5	F	G1	0	0	0	30
Bom Jesus	5	F	G1	0	0	0	30
Bom Jesus	6	M	G1	0	0	0	30
Bom Jesus	6	M	G1	0	0	0	30
Bom Jesus	6	F	G1	0	0	0	30
Bom Jesus	6	M	G1	0	0	0	30
Bom Jesus	6	F	G1	0	0	0	30
Bom Jesus	6	F	G1	0	0	0	30
Bom Jesus	7	F	G1	0	0	0	30
Bom Jesus	7	F	G2	0	0	0	30
Bom Jesus	7	M	G1	0	0	0	30
Bom Jesus	7	M	G1	0	0	0	30
Bom Jesus	7	F	G1	0	0	0	30
Bom Jesus	8	F	G1	0	0	0	30
Bom Jesus	8	M	G1	0	0	0	16
Bom Jesus	8	F	G2	0	0	0	30
Bom Jesus	8	M	G1	0	0	0	30
Bom Jesus	8	F	G1	0	0	0	30
Bom Jesus	8	M	G1	0	0	0	30
Bom Jesus	8	M	G2	0	0	0	30
Bom Jesus	8	F	G1	0	0	0	30
Bom Jesus	8	M	G1	0	0	0	30
Bom Jesus	8	M	G1	0	0	0	30
Bom Jesus	8	M	G1	0	0	0	30
Bom Jesus	9	M	G1	0	0	0	30
Bom Jesus	9	M	G1	0	0	0	30
Bom Jesus	9	F	G2	0	0	0	30
Bom Jesus	9	F	G1	0	0	0	30
Bom Jesus	9	M	G1	0	0	0	30
Bom Jesus	9	M	G1	0	0	0	30
Bom Jesus	9	F	G1	0	0	0	30
Bom Jesus	10	M	G2	0	0	0	30

Bom Jesus	10	M	G1	0	0	0	30
Bom Jesus	10	M	G1	0	0	0	30
Bom Jesus	10	F	G2	0	0	0	30
Bom Jesus	10	M	G1	0	0	0	30
Bom Jesus	10		G1	0	0	0	30
Bom Jesus	11	M	G1	0	0	0	30
Bom Jesus	11	F	G2	0	0	0	30
Bom Jesus	11	M	G1	0	0	0	30
Bom Jesus	11	F	G1	0	0	0	30
Bom Jesus	11	F	G1	0	0	0	30
Bom Jesus	12	M	G1	0	0	0	30
Bom Jesus	12	M	G1	0	0	0	30
Bom Jesus	12	M	G1	0	0	0	30
Bom Jesus	12	F	G2	0	0	0	30
Bom Jesus	13	M	G2	0	0	0	30
Bom Jesus	13	M	G2	0	0	0	30
Bom Jesus	13	M	G1	0	0	0	30
Bom Jesus	13	M	G1	0	0	0	30
Bom Jesus	13	M	G1	0	0	0	30
Bom Jesus	13	M	G3	0	0	0	30
Bom Jesus	13	F	G2	0	0	0	30
Bom Jesus	14	F	G1	0	0	0	30
Bom Jesus	14	M	G1	0	0	0	30
Bom Jesus	14	F	G1	0	0	0	30
Bom Jesus	14	M	G1	0	0	0	30
Bom Jesus	14	M	G1	0	0	0	30
Bom Jesus	14	M	G1	0	0	0	30
Bom Jesus	15	F	G1	0	0	0	30
Bom Jesus	15	M	G1	0	0	0	30
Bom Jesus	15	F	G3	0	0	0	30
Bom Jesus	16	M	G1	0	0	0	30
Bom Jesus	16	F	G1	0	0	0	30
Bom Jesus	16	M	G1	0	0	0	30
Bom Jesus	16	F	G1	0	0	0	30
Bom Jesus	16	F	G1	0	0	0	30
Bom Jesus	16	F	G1	0	0	0	30
Bom Jesus	16	F	G1	0	0	0	30
Bom Jesus	16	F	G2	0	0	0	30
Bom Jesus	17	F	G1	0	0	0	30
Bom Jesus	17	F	G1	0	0	0	30
Bom Jesus	17	M	G1	0	0	0	30
Bom Jesus	17	M	G1	0	0	0	30
Bom Jesus	17	F	G1	0	0	0	30
Bom Jesus	18	F	G1	0	0	0	30
Bom Jesus	18	M	G1	0	0	0	30
Bom Jesus	18	M	G1	0	0	0	30
Bom Jesus	18	M	G1	0	0	0	30
Bom Jesus	18	F	G2	8	11	1115	30
Bom Jesus	18	F	G2	0	0	0	30
Bom Jesus	19	M	G1	0	0	0	30

Bom Jesus	19	F	G1	0	0	0	30
Bom Jesus	19	M	G1	0	0	0	30
Bom Jesus	19	M	G1	0	0	0	30
Bom Jesus	19	M	G1	0	0	0	30
Bom Jesus	19	M	G1	0	0	0	30
Bom Jesus	20	F	G1	0	0	0	30
Bom Jesus	20	M	G1	0	0	0	30
Bom Jesus	20	M	G1	0	0	0	30
Bom Jesus	20	M	G1	0	0	0	30
Bom Jesus	21	F	G1	0	0	0	30
Bom Jesus	22	F	G2	0	0	0	30
Bom Jesus	22	F	G2	0	0	0	30
Bom Jesus	22	F	G1	0	0	0	30
Bom Jesus	23	F	G1	0	0	0	30
Bom Jesus	23	M	G1	0	0	0	30
Bom Jesus	23	M	G1	0	0	0	30
Bom Jesus	23	M	G1	0	0	0	30
Bom Jesus	24	F	G1	0	0	0	30
Bom Jesus	24	F	G1	0	0	0	12
Bom Jesus	24	F	G1	0	0	0	30
Bom Jesus	24	M	G1	0	0	0	30
Bom Jesus	24	F	G1	0	0	0	30
Bom Jesus	24	M	G1	0	0	0	30
Bom Jesus	24	M	G1	0	0	0	30
Bom Jesus	24	M	G1	0	0	0	30
Bom Jesus	25	F	G1	0	0	0	30
Bom Jesus	25	F	G3	0	0	0	30
Bom Jesus	26	F	G3	0	0	0	30
Bom Jesus	26	F	G1	0	0	0	30
Bom Jesus	26	F	G1	0	0	0	30
Bom Jesus	26	M	G1	0	0	0	30
Bom Jesus	27	F	G3	0	0	0	30
Bom Jesus	27	M	G1	0	0	0	30
Bom Jesus	27	F	G2	0	0	0	30
Bom Jesus	28	F	G1	0	0	0	30
Bom Jesus	28	F	G1	0	0	0	30
Bom Jesus	28	M	G1	0	0	0	30
Bom Jesus	29	F	G1	0	0	0	30
Bom Jesus	29	M	G1	0	0	0	30
Bom Jesus	29	M	G1	0	0	0	30
Bom Jesus	29	M	G1	0	0	0	30
Bom Jesus	30	M	G1	0	0	0	30
Bom Jesus	30	M	G1	0	0	0	30
Bom Jesus	30	M	G1	0	0	0	30
Bom Jesus	30	F	G1	0	0	0	30
Bom Jesus	30	F	G1	0	0	0	30
Bom Jesus	31	M	G1	0	0	0	30
Bom Jesus	31	M	G1	0	0	0	30
Bom Jesus	32	F	G1	0	0	0	30
Bom Jesus	32	M	G1	0	0	0	30

Bom Jesus	32	F	G2	0	0	0	30
Bom Jesus	32	M	G1	0	0	0	30
Bom Jesus	32	F	G1	0	0	0	30
Bom Jesus	33	M	G1	0	0	0	30
Bom Jesus	33	M	G1	0	0	0	30
Bom Jesus	33	F	G2	0	0	0	30
Bom Jesus	33	F	G1	0	0	0	30
Bom Jesus	34	F	G1	0	0	0	30
Bom Jesus	34	M	G1	0	0	0	30
Bom Jesus	35	F	G1	0	0	0	30
Bom Jesus	35	F	G1	0	0	0	30
Bom Jesus	36	M	G1	0	0	0	30
Bom Jesus	36	F	G2	0	0	0	30
Bom Jesus	36	F	G1	0	0	0	30
Bom Jesus	36	F	G1	0	0	0	30
Bom Jesus	37	M	G1	0	0	0	30
Bom Jesus	37	F	G1	0	0	0	30
Bom Jesus	37	M	G1	0	0	0	30
Bom Jesus	38	M	G1	0	0	0	30
Bom Jesus	38	M	G1	0	0	0	30
Bom Jesus	38	M	G1	0	0	0	30
Bom Jesus	38	F	G1	0	0	0	30
Bom Jesus	38	F	G1	0	0	0	30
Bom Jesus	38	F	G1	0	0	0	30
Bom Jesus	38	M	G2	0	0	0	30
Bom Jesus	39	F	G1	0	0	0	30
Bom Jesus	39	M	G1	0	0	0	30
Bom Jesus	39	F	G1	0	0	0	30
Bom Jesus	39	M	G1	0	0	0	30
Bom Jesus	39	F	G1	0	0	0	30
Bom Jesus	40	F	G1	0	0	0	30
Bom Jesus	40	F	G1	0	0	0	30
Bom Jesus	40	M	G1	0	0	0	30
Bom Jesus	40	F	G1	0	0	0	30
Bom Jesus	40	M	G1	0	0	0	30
Bom Jesus	41	M	G1	0	0	0	30
Bom Jesus	41	M	G1	0	0	0	30
Bom Jesus	41	F	G1	0	0	0	30
Bom Jesus	41	M	G1	0	0	0	30
Bom Jesus	41	F	G1	0	0	0	30
Bom Jesus	41	F	G1	0	0	0	30
Bom Jesus	42	F	G1	0	0	0	30
Bom Jesus	42	F	G1	0	0	0	30
Bom Jesus	43	M	G1	0	0	0	30
Bom Jesus	43	F	G1	0	0	0	30
Bom Jesus	43	F	G1	0	0	0	30
Bom Jesus	44	F	G1	0	0	0	30
Bom Jesus	44	F	G1	0	0	0	30
Bom Jesus	44	F	G1	0	0	0	30
Bom Jesus	44	F	G1	0	0	0	30

Bom Jesus	45	M	G1	0	0	0	30
Bom Jesus	46	F	G1	0	0	0	30
Bom Jesus	47	M	G1	0	0	0	30
Bom Jesus	47	F	G1	0	0	0	30
Bom Jesus	47	M	G1	0	0	0	30
Bom Jesus	47	F	G1	0	0	0	30
Bom Jesus	48	F	G1	0	0	0	30
Bom Jesus	48	M	G1	0	0	0	30
Bom Jesus	49	M	G1	0	0	0	30
Bom Jesus	50	F	G1	0	0	0	30
Bom Jesus	51	F	G1	0	0	0	30
Bom Jesus	51	M	G1	0	0	0	30
Bom Jesus	51	F	G1	0	0	0	30
Bom Jesus	52	M	G1	0	0	0	30
Bom Jesus	52	F	G1	0	0	0	30
Bom Jesus	53	F	G1	0	0	0	30
Bom Jesus	53	M	G1	0	0	0	30
Bom Jesus	54	M	G1	0	0	0	30
Bom Jesus	56	M	G1	0	0	0	30
Bom Jesus	56	F	G2	0	0	0	30
Bom Jesus	57	M	G1	0	0	0	30
Bom Jesus	57	M	G1	0	0	0	30
Bom Jesus	58	F	G1	0	0	0	30
Bom Jesus	60	M	G2	0	0	0	30
Bom Jesus	61	F	G1	0	0	0	30
Bom Jesus	61	F	G1	0	0	0	30
Bom Jesus	63	M	G1	0	0	0	30
Bom Jesus	64	F	G1	0	0	0	30
Bom Jesus	64	M	G1	0	0	0	30
Bom Jesus	65	F	G1	0	0	0	30
Bom Jesus	67	M	G1	0	0	0	30
Bom Jesus	68	M	G1	0	0	0	30
Bom Jesus	68	F	G1	0	0	0	30
Bom Jesus	70	F	G1	0	0	0	30
Bom Jesus	71	F	G1	0	0	0	30
Bom Jesus	71	F	G1	0	0	0	30
Bom Jesus	72	F	G1	0	0	0	30
Bom Jesus	73	F	G1	0	0	0	30
Bom Jesus	74	F	G1	0	0	0	30
Bom Jesus	74	F	G1	0	0	0	30
Bom Jesus	75	F	G1	0	0	0	30
Bom Jesus	75	M	G1	0	0	0	30
Bom Jesus	76	F	G1	0	0	0	30
Bom Jesus	78	M	G1	0	0	0	30
Bom Jesus	79	F	G1	0	0	0	30
Bom Jesus	83	F	G1	0	0	0	30
Bom Jesus	88	M	G1	0	0	0	30
Bom Jesus	98	F	G2	0	0	0	30
Palmital	3	F	G1	0	0	0	30
Palmital	4	F	G1	0	0	0	30

Palmital	4	M	G1	0	0	0	30
Palmital	5	F	G1	0	0	0	30
Palmital	5	M	G1	0	0	0	30
Palmital	7	F	G1	0	0	0	30
Palmital	8	F	G1	0	0	0	30
Palmital	9	M	G1	0	0	0	30
Palmital	10	M	G1	0	0	0	30
Palmital	10	F	G1	0	0	0	30
Palmital	10	F	G1	0	0	0	30
Palmital	10	F	G1	0	0	0	30
Palmital	10	F	G1	0	0	0	30
Palmital	11	F	G1	0	0	0	30
Palmital	14	M	G1	0	0	0	30
Palmital	14	M	G1	0	0	0	30
Palmital	14	M	G1	0	0	0	30
Palmital	14	F	G1	0	0	0	30
Palmital	14	F	G1	0	0	0	30
Palmital	15	M	G1	0	0	0	30
Palmital	16	M	G1	0	0	0	30
Palmital	17	F	G1	0	0	0	30
Palmital	17	F	G1	0	0	0	30
Palmital	18	F	G1	0	0	0	30
Palmital	18	M	G1	0	0	0	30
Palmital	19	F	G1	0	0	0	30
Palmital	20	M	G1	0	0	0	30
Palmital	21	F	G1	0	0	0	30
Palmital	22	F	G1	0	0	0	30
Palmital	23	F	G1	0	0	0	30
Palmital	23	F	G1	0	0	0	30
Palmital	23	F	G1	0	0	0	30
Palmital	24	F	G1	0	0	0	30
Palmital	25	M	G1	0	0	0	30
Palmital	26	M	G1	0	0	0	30
Palmital	26	M	G1	0	0	3	30
Palmital	28	F	G1	0	0	0	30
Palmital	28	F	G1	0	0	0	30
Palmital	28	M	G1	0	0	0	30
Palmital	28	F	G1	0	0	3	30
Palmital	28	F	G1	0	0	0	30
Palmital	30	M	G1	0	0	0	30
Palmital	30	M	G1	0	0	0	30
Palmital	30	M	G1	0	0	0	30
Palmital	31	M	G4	1	2	2	30
Palmital	33	F	G1	0	0	0	30
Palmital	33	F	G1	0	0	0	30
Palmital	35	F	G1	0	0	0	30
Palmital	38	F	G1	0	0	0	30
Palmital	38	M	G1	0	0	0	30
Palmital	38	M	G1	0	0	0	30
Palmital	38	M	G1	0	0	0	30

Palmital	39	F	G1	0	0	0	30
Palmital	40	F	G1	0	0	0	30
Palmital	40	M	G1	0	0	0	30
Palmital	40	M	G1	0	0	0	30
Palmital	41	F	G1	0	0	0	30
Palmital	42	F	G1	0	0	0	30
Palmital	42	M	G1	0	0	0	30
Palmital	47	M	G1	0	0	0	30
Palmital	48	F	G1	0	0	0	30
Palmital	49	F	G1	0	0	0	30
Palmital	51	M	G1	0	0	0	30
Palmital	51	M	G1	0	0	0	30
Palmital	52	M	G1	0	0	0	30
Palmital	52	F	G1	0	0	0	30
Palmital	52	F	G1	0	0	0	30
Palmital	53	M	G1	0	0	0	30
Palmital	53	M	G1	0	0	0	30
Palmital	54	M	G1	0	0	0	30
Palmital	55	M	G1	0	0	0	30
Palmital	56	M	G1	0	0	0	30
Palmital	56	F	G1	0	0	0	30
Palmital	56	M	G1	0	0	0	30
Palmital	57	M	G1	0	0	0	30
Palmital	57	M	G1	0	0	0	30
Palmital	57	F	G1	0	0	0	30
Palmital	58	M	G1	0	0	0	30
Palmital	58	M	G1	0	0	0	30
Palmital	59	M	G1	0	0	1	30
Palmital	61	F	G1	0	0	0	30
Palmital	62	F	G1	0	0	0	30
Palmital	62	M	G1	0	0	0	30
Palmital	62	M	G4	0	0	0	30
Palmital	62	F	G1	0	0	0	30
Palmital	63	F	G1	0	0	0	30
Palmital	65	F	G1	0	0	0	30
Palmital	65	M	G1	0	0	0	30
Palmital	66	F	G1	0	0	0	30
Palmital	67	F	G1	0	0	0	30
Palmital	67	M	G1	0	0	0	30
Palmital	68	M	G1	0	0	0	30
Palmital	68	M	G1	0	0	0	30
Palmital	72	F	G1	0	0	0	30
Palmital	74	F	G1	0	0	0	30
Palmital	74	M	G1	0	0	0	30
Palmital	82	F	G1	0	0	0	30
Est Miralta	2	F	G9	0	0	0	30
Est Miralta	3	F	G3	0	0	0	30
Est Miralta	5	M	G6	0	0	0	30
Est Miralta	6	F	G1	0	0	0	30
Est Miralta	8	F	G4	0	0	0	30

Est Miralta	8	F	G1	0	0	0	30
Est Miralta	8	F	G9	0	0	0	30
Est Miralta	8	M	G1	0	0	0	30
Est Miralta	8	F	G1	0	0	0	30
Est Miralta	9	M	G1	0	0	0	30
Est Miralta	10	M	G1	0	0	0	30
Est Miralta	10	M	G1	0	0	0	30
Est Miralta	11	M	G1	0	0	0	30
Est Miralta	11	M	G3	0	0	0	30
Est Miralta	11	F	G2	0	0	0	30
Est Miralta	12	M	G5	0	0	0	30
Est Miralta	13	M	G5	0	0	0	30
Est Miralta	13	F	G2	0	0	0	30
Est Miralta	14	M	G1	0	0	0	30
Est Miralta	15	M	G1	0	0	0	30
Est Miralta	15	M	G1	0	0	0	30
Est Miralta	16	F	G1	0	0	0	30
Est Miralta	16	M	G1	0	0	0	30
Est Miralta	18	M	G1	0	0	0	30
Est Miralta	18	M	G2	0	0	0	30
Est Miralta	19	F	G1	0	0	0	30
Est Miralta	20	M	G1	0	0	0	30
Est Miralta	22	M	G1	0	0	0	30
Est Miralta	25	F	G2	0	0	0	30
Est Miralta	26	F	G2	0	0	0	30
Est Miralta	29	F	G2	0	0	0	30
Est Miralta	30	M	G2	0	2	16	30
Est Miralta	31	F	G2	0	0	0	30
Est Miralta	32	M	G1	0	0	0	30
Est Miralta	33	M	G5	1	1	3	30
Est Miralta	33	F	G1	0	0	0	30
Est Miralta	33	M	G1	0	0	0	30
Est Miralta	34	M	G1	0	0	0	30
Est Miralta	34	M	G1	0	0	0	30
Est Miralta	35	F	G1	0	0	0	30
Est Miralta	36	F	G2	0	0	0	30
Est Miralta	36	M	G1	0	0	0	30
Est Miralta	36	F	G1	0	0	0	30
Est Miralta	37	F	G4	0	0	0	30
Est Miralta	37	F	G5	0	0	0	30
Est Miralta	38	F	G6	0	0	0	30
Est Miralta	38	F	G1	0	0	0	30
Est Miralta	38	M	G2	0	0	0	30
Est Miralta	38	F	G1	0	0	0	30
Est Miralta	39	F	G3	0	0	0	30
Est Miralta	39	F	G2	0	0	0	30
Est Miralta	39	M	G4	0	0	0	30
Est Miralta	41	F	G1	0	0	0	30
Est Miralta	42	F	G5	0	0	0	30
Est Miralta	43	M	G5	0	0	0	30

Est Miralta	43	M	G1	0	0	0	30
Est Miralta	43	M	G1	0	0	0	30
Est Miralta	44	M	G5	0	0	0	30
Est Miralta	45	M	G1	0	0	0	30
Est Miralta	46	F	G3	0	0	0	30
Est Miralta	47	F	G1	0	0	0	30
Est Miralta	48	M	G1	0	0	0	30
Est Miralta	48	M	G1	0	0	0	30
Est Miralta	48	F	G1	0	0	0	30
Est Miralta	49	F	G1	0	0	0	30
Est Miralta	49	M	G2	0	0	0	30
Est Miralta	49	M	G1	0	0	0	30
Est Miralta	49	M	G10	0	0	0	30
Est Miralta	50	F	G1	0	0	0	30
Est Miralta	51	F	G2	0	0	0	30
Est Miralta	51	M	G2	0	0	0	30
Est Miralta	52	F	G1	0	0	0	30
Est Miralta	52	F	G2	0	0	0	30
Est Miralta	53	F	G1	0	0	0	30
Est Miralta	53	F	G1	0	0	0	30
Est Miralta	54	M	G1	0	0	0	30
Est Miralta	54	F	G1	0	0	0	30
Est Miralta	56	F	G2	0	0	0	30
Est Miralta	58	M	G1	0	0	0	30
Est Miralta	60	M	G1	0	0	0	30
Est Miralta	60	M	G1	0	0	0	30
Est Miralta	61	F	G2	0	0	0	30
Est Miralta	62	F	G2	0	0	0	30
Est Miralta	63	M	G3	0	0	0	30
Est Miralta	63	M	G2	0	0	0	30
Est Miralta	66	F	G1	0	0	0	30
Est Miralta	66	M	G4	0	0	0	30
Est Miralta	69	F	G1	0	0	0	30
Est Miralta	71	F	G1	0	0	0	30
Est Miralta	71	M	G3	0	0	0	30
Est Miralta	71	M	G1	0	0	0	30
Est Miralta	72	M	G1	0	0	0	30
Est Miralta	73	F	G1	0	0	0	30
Est Miralta	73	M	G6	0	0	0	30
Est Miralta	82	F	G1	0	0	0	30
Est Miralta	83	F	G1	0	0	0	30
Est Miralta	83	M	G1	0	0	0	30
Est Miralta	83	F	G1	0	0	0	30
Gavião	3	F	G6	0	0	0	30
Gavião	3	M	G2	0	0	0	30
Gavião	3	M	G2	0	0	0	30
Gavião	4	F	G7	0	0	0	30
Gavião	5	F	G5	0	0	0	30
Gavião	5	F	G4	0	0	0	30
Gavião	6	F	G3	0	0	0	30

Gavião	6	M	G5	0	0	0	30
Gavião	6	F	G6	0	0	0	30
Gavião	6	F	G1	0	0	0	30
Gavião	6	M	G4	0	0	0	30
Gavião	7	M	G5	0	0	0	30
Gavião	7	M	G2	0	0	0	30
Gavião	7	F	G5	0	0	1	30
Gavião	7	M	G5	0	0	0	30
Gavião	7	F	G5	0	0	0	30
Gavião	7	M	G3	0	0	0	30
Gavião	8	F	G3	0	0	0	30
Gavião	8	F	G3	0	0	0	30
Gavião	8	F	G5	0	0	0	30
Gavião	8	M	G5	0	0	0	30
Gavião	8	M	G1	0	0	0	30
Gavião	9	F	G4	0	0	0	30
Gavião	9	F	G2	0	0	0	30
Gavião	9	M	G1	0	0	0	30
Gavião	9	F	G3	0	0	0	30
Gavião	9	F	G1	0	0	0	30
Gavião	9	M	G3	0	0	0	30
Gavião	9	M	G1	0	0	0	30
Gavião	10	M	G7	0	0	0	30
Gavião	10	F	G4	0	0	0	30
Gavião	11	M	G6	0	0	0	30
Gavião	12	F	G4	0	0	0	30
Gavião	12	M	G4	0	0	0	30
Gavião	12	F	G1	0	0	0	30
Gavião	12	M	G3	0	0	0	30
Gavião	13	M	G6	0	0	1	30
Gavião	13	F	G4	0	0	3	30
Gavião	14	F	G2	0	0	0	30
Gavião	14	F	G3	0	0	0	30
Gavião	15	F	G3	0	0	0	30
Gavião	16	M	G1	0	0	0	30
Gavião	16	M	G5	0	0	0	30
Gavião	16	F	G2	0	0	0	30
Gavião	17	M	G3	0	0	0	30
Gavião	17	F	G1	0	0	0	30
Gavião	18	F	G2	0	0	0	30
Gavião	18	F	G2	0	0	0	30
Gavião	18	M	G5	0	0	1	30
Gavião	18	F	G4	0	0	0	30
Gavião	19	F	G3	0	0	0	30
Gavião	20	M	G2	0	0	0	30
Gavião	20	M	G1	0	0	0	30
Gavião	20	F	G2	0	0	0	30
Gavião	20	M	G4	0	0	0	30
Gavião	21	F	G4	0	0	0	30
Gavião	21	M	G1	0	0	0	30

Gavião	22	M	G3	0	0	0	30
Gavião	23	F	G2	0	0	0	30
Gavião	23	F	G2	0	0	0	30
Gavião	24	M	G1	0	0	0	30
Gavião	24	F	G7	0	0	0	30
Gavião	26	F	G5	0	0	0	30
Gavião	26	M	G3	0	0	0	30
Gavião	26	F	G3	0	1	1	30
Gavião	27	M	G3	0	0	0	30
Gavião	28	M	G5	0	0	0	30
Gavião	28	F	G4	0	0	0	30
Gavião	28	F	G2	0	0	0	30
Gavião	28	M	G2	0	0	0	30
Gavião	29	F	G3	0	0	0	30
Gavião	30	F	G1	0	0	0	30
Gavião	30	F	G2	0	0	0	30
Gavião	30	F	G3	0	0	1	30
Gavião	31	F	G1	0	0	0	30
Gavião	31	M	G1	0	0	1	30
Gavião	31	M	G2	0	0	0	30
Gavião	32	F	G1	0	0	0	30
Gavião	32	M	G1	0	0	0	30
Gavião	33	F	G2	0	0	0	30
Gavião	33	F	G3	0	0	0	30
Gavião	33	M	G1	0	0	0	30
Gavião	33	M	G4	0	0	0	30
Gavião	33	F	G3	0	0	0	30
Gavião	33	F	G1	0	0	0	30
Gavião	34	F	G1	0	0	0	30
Gavião	34	F	G2	0	0	0	30
Gavião	34	M	G1	0	0	0	30
Gavião	35	M	G7	0	0	0	30
Gavião	35	F	G2	0	0	0	30
Gavião	35	F	G4	0	0	0	30
Gavião	35	F	G3	1	1	2	30
Gavião	36	M	G2	0	0	0	30
Gavião	36	F	G4	0	0	0	30
Gavião	36	F	G3	0	0	0	30
Gavião	36	M	G1	0	0	0	30
Gavião	36	F	G2	0	0	1	30
Gavião	36	F	G1	0	0	0	30
Gavião	36	M	G2	0	0	0	30
Gavião	36	F	G1	0	0	0	30
Gavião	36	F	G2	0	0	0	30
Gavião	37	M	G2	0	0	0	30
Gavião	37	F	G3	0	0	0	30
Gavião	38	F	G2	0	0	0	30
Gavião	38	F	G2	0	0	0	30
Gavião	38	F	G1	0	0	0	30
Gavião	38	M	G2	0	0	0	30

Gavião	39	M	G2	0	0	0	30
Gavião	39	F	G4	1	1	1	30
Gavião	39	F	G1	0	0	0	30
Gavião	40	F	G2	0	0	0	30
Gavião	40	M	G2	0	0	0	30
Gavião	40	F	G2	0	0	0	30
Gavião	40	F	G4	0	0	0	30
Gavião	41	M	G2	0	0	0	30
Gavião	41	F	G3	0	0	0	30
Gavião	41	M	G2	0	0	0	30
Gavião	42	F	G4	0	0	0	30
Gavião	42	F	G3	0	0	0	30
Gavião	42	F	G2	0	0	0	30
Gavião	42	M	G1	0	0	0	30
Gavião	42	M	G2	0	0	0	30
Gavião	43	M	G4	0	0	0	30
Gavião	43	F	G5	0	1	3	30
Gavião	43	F	G5	0	0	6	30
Gavião	43	M	G3	0	0	0	30
Gavião	43	F	G1	0	0	0	30
Gavião	43	M	G1	0	0	0	30
Gavião	43	F	G1	0	0	0	30
Gavião	43	F	G1	0	0	0	30
Gavião	44	F	G2	0	0	0	30
Gavião	44	M	G3	0	0	0	30
Gavião	44	M	G3	0	0	0	30
Gavião	45	F	G1	0	0	0	30
Gavião	45	M	G2	0	0	0	30
Gavião	45	F	G2	0	0	0	30
Gavião	45	M	G1	0	0	0	30
Gavião	46	M	G1	0	0	0	30
Gavião	46	M	G1	0	0	0	30
Gavião	46	F	G2	0	0	0	30
Gavião	46	F	G1	0	0	0	30
Gavião	47	M	G1	0	0	0	30
Gavião	47	M	G1	0	0	0	30
Gavião	48	F	G6	0	0	0	30
Gavião	48	M	G2	0	0	0	30
Gavião	48	M	G2	0	0	0	30
Gavião	49	F	G2	0	0	0	30
Gavião	49	M	G3	0	0	0	30
Gavião	50	M	G2	0	0	0	30
Gavião	50	F	G1	0	0	0	30
Gavião	50	M	G1	0	0	0	30
Gavião	50	F	G1	0	0	0	30
Gavião	51	F	G3	0	0	0	30
Gavião	51	M	G3	0	0	0	30
Gavião	51	M	G3	0	0	0	30
Gavião	51	F	G1	0	0	0	30
Gavião	51	F	G1	0	0	0	30

Gavião	51	F	G2	0	1	2	30
Gavião	51	F	G2	0	0	0	30
Gavião	51	F	G1	0	0	0	30
Gavião	52	M	G1	0	0	0	30
Gavião	53	F	G1	0	0	0	30
Gavião	53	F	G1	0	0	0	30
Gavião	54	M	G2	0	0	0	30
Gavião	54	M	G2	0	1	0	30
Gavião	54	M	G1	0	0	0	30
Gavião	54	F	G2	0	0	0	30
Gavião	54	M	G1	0	0	0	30
Gavião	54	M	G1	0	0	0	30
Gavião	55	F	G1	0	0	0	30
Gavião	55	F	G5	0	0	0	30
Gavião	55	M	G3	0	0	0	30
Gavião	55	F	G2	0	0	0	30
Gavião	55	F	G3	0	0	0	30
Gavião	55	M	G4	0	0	0	30
Gavião	57	M	G3	0	0	0	30
Gavião	57	M	G2	0	0	0	30
Gavião	57	M	G1	0	0	0	30
Gavião	58	F	G2	0	0	0	30
Gavião	59	F	G1	0	0	0	30
Gavião	60	F	G4	0	0	0	30
Gavião	63	M	G1	0	0	0	30
Gavião	63	M	G3	0	0	0	30
Gavião	63	F	G3	0	0	0	30
Gavião	64	F	G1	0	0	0	30
Gavião	66	M	G2	0	0	0	30
Gavião	66	F	G2	0	0	0	30
Gavião	67	F	G1	0	0	0	30
Gavião	69	M	G3	0	0	0	30
Gavião	69	F	G2	0	0	1	30
Gavião	70	F	G1	0	0	0	30
Gavião	70	F	G2	0	0	0	30
Gavião	71	F	G2	0	0	0	30
Gavião	72	F	G1	0	0	0	30
Gavião	72	M	G4	0	0	0	30
Gavião	72	M	G1	0	0	0	30
Gavião	73	F	G3	0	0	0	30
Gavião	73	F	G2	0	0	0	30
Gavião	73	F	G4	0	0	1	30
Gavião	73	F	G2	0	0	0	30
Gavião	75	F	G3	0	0	0	30
Gavião	75	F	G1	0	0	0	30
Gavião	76	F	G1	0	0	0	30
Gavião	76	F	G1	0	0	0	30
Gavião	77	M	G1	0	0	0	30
Gavião	78	M	G2	0	0	0	30
Gavião	80	M	G4	0	0	0	30

Gavião	80	F	G3	0	0	0	30
Gavião	80	M	G1	0	0	0	30
Gavião	81	F	G2	0	0	1	30
Gavião	82	F	G1	0	0	0	30
Gavião	86	M	G4	0	0	0	30
Gavião	87	F	G2	0	0	0	30
Gavião	87	F	G4	0	0	0	30
Gavião	87	M	G5	0	0	0	30
Gavião	.	F	G2	0	0	0	30
Gavião	.	M	G1	0	0	0	30
Gavião	.	F	G1	0	0	0	30
Gavião	.	F	G1	0	0	0	30
Gavião	.	M	G1	0	0	0	30
Gavião	.	M	G2	0	0	0	30
Gavião	.	M	G1	0	0	0	30
Gavião	.	F	G3	0	0	0	30
Gavião	.	F	G1	0	0	0	30
Gavião	.	M	G1	0	0	0	30
Gavião	.	F	G2	0	0	0	30
Gavião	.	M	G1	0	0	0	30
Gavião	.	M	G1	0	0	0	30
Gavião	.	M	G1	0	0	0	30
Gavião	.	M	G1	0	0	0	30
Gavião	.	F	G4	0	0	0	30
Gavião	.	F	G1	0	0	0	30
Gavião	.	M	G2	0	0	0	30
Gavião	.	F	G2	0	0	0	30
Gavião	.	M	G4	0	0	0	30
Gavião	.	M	G3	0	0	0	30
Gavião	.	F	G1	0	0	0	30
Gavião	.	M	G1	0	0	0	30
Gavião	.	F	G3	0	0	2	30
Gavião	.	F	G3	0	0	0	30
Gavião	.	M	G4	0	0	0	30
Gavião	.	F	G5	0	0	0	30
Gavião	.	F	G4	0	0	0	30
Gavião	.	M	G1	0	0	0	30
Gavião	.	F	G4	0	0	0	30
Gavião	.	M	G1	0	0	0	30
Gavião	.	M	G3	0	0	0	30
Gavião	.	F	G4	0	0	0	30
Gavião	.	F	G2	0	0	0	30
Gavião	.	F	G5	0	0	0	30
Gavião	.	F	G9	0	0	0	30
Gavião	.	F	G4	0	0	0	30
Gavião	.	M	G3	0	0	0	30
Gavião	.	M	G2	0	0	0	30
Gavião	.	M	G4	0	0	0	30
Gavião	.	M	G1	0	0	0	30
Gavião	.	M	G1	0	0	0	30

Gavião	.	M	G2	0	0	0	30
Gavião	.	M	G1	0	0	2	30
Gavião	.	M	G2	0	0	0	30
Gavião	.	F	G1	0	0	0	30
Gavião	.	F	G1	0	0	0	30
Gavião	.	F	G10	0	0	0	30
Gavião	.	M	G1	0	0	0	30
Gavião	.	F	G5	0	0	0	30
Gavião	.	M	G2	0	0	0	30
Gavião	.	F	G1	0	0	0	30
Gavião	.	F	G4	0	0	0	30
Gavião	.	M	G5	0	0	0	30
Gavião	.	F	G4	0	0	0	30
Gavião	.	F	G4	0	0	1	30
Gavião	.	F	G6	0	1	1	30
Gavião	.	F	G1	0	0	0	30
Gavião	.	F	G6	0	0	0	30
Gavião	.	M	G2	0	0	0	30
Gavião	.	M	G3	0	0	2	30
Gavião	.	F	G5	0	0	0	30
Gavião	.	M	G3	0	0	4	30
Gavião	.		G4	0	0	0	30
Gavião	.		G5	0	0	0	30
Gavião	.	F	G2	0	0	0	30
Gavião	.	F	G5	0	0	0	30
Gavião	.	M	G2	0	0	0	30
Gavião	.	M	G3	0	0	0	30
Gavião	.	F	G2	0	0	0	30
Gavião	.	F	G2	0	0	0	30
Itaquara	4	M	G1	0	0	0	30
Itaquara	5	F	G5	0	0	0	30
Itaquara	5	F	G3	0	0	0	30
Itaquara	5	F	G3	0	0	0	30
Itaquara	5	M	G1	0	0	0	30
Itaquara	6	M	G1	0	0	0	30
Itaquara	6	M	G1	0	0	0	30
Itaquara	6	M	G5	0	0	0	30
Itaquara	6	F	G1	0	0	0	30
Itaquara	6	F	G1	0	0	0	30
Itaquara	6	M	G4	0	0	1	30
Itaquara	6	F	G1	0	0	0	30
Itaquara	7	M	G3	0	0	1	30
Itaquara	7	F	G3	0	0	2	30
Itaquara	8	F	G1	0	0	0	30
Itaquara	8	M	G1	0	0	0	30
Itaquara	9	M	G2	0	0	3	30
Itaquara	9	M	G3	0	0	0	30
Itaquara	9	M	G3	0	0	0	30
Itaquara	9	M	G1	0	0	0	30
Itaquara	9	F	G3	0	0	1	30

Itaquara	10	M	G1	0	0	0	30
Itaquara	10	F	G6	0	0	0	30
Itaquara	11	F	G1	0	0	0	30
Itaquara	11	F	G3	0	0	0	30
Itaquara	11	M	G1	0	0	0	30
Itaquara	11	M	G3	0	0	1	30
Itaquara	11	M	G2	0	0	0	30
Itaquara	11	M	G3	0	0	0	30
Itaquara	12	F	G2	0	0	1	30
Itaquara	12	M	G1	0	0	0	30
Itaquara	12	M	G1	0	0	0	30
Itaquara	13	M	G3	0	0	0	30
Itaquara	13	F	G1	0	0	0	30
Itaquara	13	M	G1	0	0	0	30
Itaquara	13	F	G5	0	0	0	30
Itaquara	14	F	G3	0	0	2	30
Itaquara	14	M	G4	0	0	0	30
Itaquara	14	M	G1	0	0	0	30
Itaquara	14	M	G1	0	0	0	30
Itaquara	15	F	G1	0	0	0	30
Itaquara	15	F	G1	0	0	0	30
Itaquara	15	F	G3	0	0	1	30
Itaquara	15	F	G5	0	0	5	30
Itaquara	15	M	G2	0	0	0	30
Itaquara	15	F	G4	0	0	0	30
Itaquara	15	M	G4	0	0	0	30
Itaquara	16	F	G1	0	0	0	30
Itaquara	16	F	G1	0	0	0	30
Itaquara	16	F	G3	0	0	0	30
Itaquara	17	M	G1	0	0	0	30
Itaquara	17	F	G1	1	1	1	30
Itaquara	17	M	G3	0	0	0	30
Itaquara	17	F	G2	0	0	0	30
Itaquara	17	F	G3	0	0	0	30
Itaquara	18	M	G2	0	0	0	30
Itaquara	18	M	G1	0	0	0	30
Itaquara	18	M	G1	0	0	0	30
Itaquara	18	F	G3	0	0	0	30
Itaquara	18	M	G4	0	0	0	30
Itaquara	18	M	G1	0	0	0	30
Itaquara	19	F	G7	0	0	0	30
Itaquara	19	M	G1	0	0	0	30
Itaquara	19	F	G6	0	0	0	30
Itaquara	19	F	G1	0	0	0	30
Itaquara	20	M	G1	0	0	0	30
Itaquara	20	F	G3	0	0	0	30
Itaquara	20	F	G1	0	0	0	30
Itaquara	21	M	G1	0	0	0	30
Itaquara	21	M	G1	0	0	0	30
Itaquara	21	M	G1	0	0	0	30

Itaquara	21	M	G1	0	0	0	30
Itaquara	21	F	G1	0	0	0	30
Itaquara	22	M	G1	0	0	0	30
Itaquara	22	M	G6	8	7	2	30
Itaquara	22	F	G1	0	0	0	30
Itaquara	23	M	G1	0	0	0	30
Itaquara	23	F	G4	0	0	1	30
Itaquara	23	M	G3	0	0	0	30
Itaquara	23	M	G3	0	0	4	30
Itaquara	24	F	G4	0	0	0	30
Itaquara	24	F	G1	2	15	36	30
Itaquara	24	M	G1	1	2	1	30
Itaquara	25	M	G1	0	0	0	30
Itaquara	25	M	G3	0	0	2	30
Itaquara	25	M	G1	1	0	0	30
Itaquara	25	M	G4	0	0	0	30
Itaquara	26	F	G1	0	0	0	30
Itaquara	26	F	G4	0	0	0	30
Itaquara	26	M	G1	0	0	0	30
Itaquara	27	F	G4	0	0	1	30
Itaquara	27	F	G1	0	0	0	30
Itaquara	28	F	G1	0	0	0	30
Itaquara	28	M	G1	0	0	0	30
Itaquara	28	F	G5	0	0	0	30
Itaquara	29	M	G1	0	0	0	30
Itaquara	29	M	G3	1	1	4	30
Itaquara	29	F	G1	0	0	0	30
Itaquara	30	M	G1	0	0	0	30
Itaquara	30	F	G1	0	0	0	30
Itaquara	30	F	G1	0	0	0	30
Itaquara	31	F	G3	0	0	1	30
Itaquara	31	M	G2	0	0	0	30
Itaquara	31	F	G1	0	0	0	30
Itaquara	32	F	G3	0	0	0	30
Itaquara	32	M	G1	0	0	0	30
Itaquara	32	M	G3	0	0	1	30
Itaquara	33	F	G1	0	0	0	30
Itaquara	33	F	G3	0	0	0	30
Itaquara	34	M	G3	0	0	1	30
Itaquara	34	M	G1	0	0	0	30
Itaquara	34	M	G3	0	0	1	30
Itaquara	35	F	G5	0	0	0	30
Itaquara	35	F	G1	0	0	0	30
Itaquara	36	F	G1	0	0	0	30
Itaquara	36	M	G3	0	0	0	30
Itaquara	36	F	G1	0	0	0	30
Itaquara	36	F	G1	0	0	0	30
Itaquara	36	M	G1	0	0	0	30
Itaquara	37	F	G3	0	0	0	30
Itaquara	37	F	G1	0	0	0	30

Itaquara	37	F	G2	0	0	0	30
Itaquara	37	F	G3	0	0	0	30
Itaquara	38	F	G1	0	0	0	30
Itaquara	38	F	G1	0	0	0	30
Itaquara	38	F	G1	0	0	0	30
Itaquara	38	M	G3	1	0	0	30
Itaquara	39	F	G7	0	0	1	30
Itaquara	39	F	G1	0	0	0	30
Itaquara	39	F	G1	0	0	0	30
Itaquara	39	M	G1	0	0	0	30
Itaquara	40	F	G1	0	0	0	30
Itaquara	40	F	G1	0	0	0	30
Itaquara	40	F	G3	0	0	5	30
Itaquara	40	M	G3	0	0	0	30
Itaquara	40	F	G1	0	0	0	30
Itaquara	40	F	G3	0	0	0	30
Itaquara	40	F	G3	0	0	0	30
Itaquara	41	F	G2	0	0	0	30
Itaquara	41	M	G3	0	0	0	30
Itaquara	41	F	G1	0	0	0	30
Itaquara	41	M	G1	0	0	0	30
Itaquara	42	F	G3	0	0	0	30
Itaquara	42	M	G1	0	0	0	30
Itaquara	42	F	G1	0	0	0	30
Itaquara	42	M	G1	0	0	0	30
Itaquara	42	F	G3	0	0	0	30
Itaquara	42	M	G3	0	0	0	30
Itaquara	42	F	G1	0	0	0	30
Itaquara	43	M	G1	0	1	0	30
Itaquara	43	M	G1	1	0	1	30
Itaquara	43	F	G1	0	0	0	30
Itaquara	43	M	G1	0	0	0	30
Itaquara	43	F	G1	0	0	0	30
Itaquara	43	F	G1	0	0	0	30
Itaquara	43	M	G1	0	0	0	30
Itaquara	44	M	G1	0	0	0	30
Itaquara	44	M	G1	0	0	0	30
Itaquara	44	M	G1	0	0	0	30
Itaquara	45	M	G3	0	0	1	30
Itaquara	45	M	G3	0	0	0	30
Itaquara	45	M	G1	0	0	0	30
Itaquara	46	M	G1	0	0	0	30
Itaquara	46	F	G1	0	0	0	30
Itaquara	46	F	G3	0	0	2	30
Itaquara	46	M	G1	0	0	0	30
Itaquara	46	M	G1	0	0	0	30
Itaquara	46	F	G2	0	0	1	30
Itaquara	46	F	G1	0	0	0	30
Itaquara	46	M	G1	0	0	0	30
Itaquara	46	M	G1	0	0	0	30

Itaquara	46	M	G2	0	0	0	30
Itaquara	46	F	G3	0	0	0	30
Itaquara	47	F	G1	0	0	0	30
Itaquara	47	F	G2	0	0	0	30
Itaquara	47	M	G1	0	0	0	30
Itaquara	47	F	G3	0	0	0	30
Itaquara	48	M	G1	0	0	0	30
Itaquara	48	F	G2	0	0	0	30
Itaquara	48	M	G1	0	0	0	30
Itaquara	48	F	G1	0	0	0	30
Itaquara	49	M	G1	0	0	0	30
Itaquara	49	M	G1	1	0	1	30
Itaquara	49	M	G1	0	0	0	30
Itaquara	49	F	G3	0	0	0	30
Itaquara	49	M	G1	0	0	0	30
Itaquara	49	M	G1	0	0	0	30
Itaquara	49	M	G1	0	0	0	30
Itaquara	50	M	G1	1	0	1	30
Itaquara	50	M	G1	0	0	0	30
Itaquara	50	F	G1	0	0	0	30
Itaquara	50	M	G3	0	0	2	30
Itaquara	50	F	G1	0	0	0	30
Itaquara	51	M	G1	0	0	0	30
Itaquara	51	M	G1	0	0	0	30
Itaquara	51	M	G3	0	0	0	30
Itaquara	51	M	G2	0	0	0	30
Itaquara	51	F	G3	0	0	0	30
Itaquara	51	F	G1	0	0	0	30
Itaquara	52	M	G4	0	0	0	30
Itaquara	52	F	G3	0	0	0	30
Itaquara	52	M	G6	0	0	3	30
Itaquara	52	M	G1	2	0	2	30
Itaquara	52	F	G4	0	0	0	30
Itaquara	52	M	G3	1	1	0	30
Itaquara	52	F	G1	0	0	0	30
Itaquara	53	M	G1	0	0	0	30
Itaquara	53	F	G2	0	0	1	30
Itaquara	54	M	G1	0	0	0	30
Itaquara	54	F	G1	0	0	1	30
Itaquara	54	M	G4	0	0	0	30
Itaquara	54	F	G3	0	0	0	30
Itaquara	54	F	G1	0	0	0	30
Itaquara	54	F	G1	0	0	0	30
Itaquara	54	M	G4	2	1	1	30
Itaquara	55	F	G5	0	0	0	30
Itaquara	55	F	G1	0	0	0	30
Itaquara	55	F	G1	0	0	0	30
Itaquara	55	F	G1	0	0	0	30
Itaquara	56	F	G4	0	0	0	30
Itaquara	56	M	G4	0	0	1	30

Itaquara	56	F	G1	0	0	0	30
Itaquara	56	F	G1	0	0	0	30
Itaquara	56	F	G1	0	0	0	30
Itaquara	57	F	G1	0	0	0	30
Itaquara	57	F	G1	0	0	0	30
Itaquara	57	F	G3	0	0	0	30
Itaquara	58	F	G3	0	0	0	30
Itaquara	59	F	G1	0	0	0	30
Itaquara	59	F	G3	0	0	0	30
Itaquara	59	F	G3	0	0	0	30
Itaquara	59	M	G1	0	0	0	30
Itaquara	59	M	G3	0	1	3	30
Itaquara	60	F	G1	0	0	0	30
Itaquara	60	M	G5	0	0	0	30
Itaquara	60	M	G1	0	0	0	30
Itaquara	61	F	G1	0	0	0	30
Itaquara	61	F	G1	0	0	0	30
Itaquara	61	M	G1	0	0	0	30
Itaquara	62	M	G3	1	3	0	30
Itaquara	62	M	G1	0	0	0	30
Itaquara	62	F	G1	0	0	0	30
Itaquara	63	M	G2	0	0	2	30
Itaquara	63	M	G1	0	0	0	30
Itaquara	63	F	G1	0	0	0	30
Itaquara	63	F	G1	0	0	0	30
Itaquara	64	M	G1	0	0	0	30
Itaquara	64	M	G1	0	0	0	30
Itaquara	65	F	G1	0	0	0	30
Itaquara	65	M	G2	0	1	2	30
Itaquara	65	M	G3	0	0	0	30
Itaquara	65	F	G1	0	0	0	30
Itaquara	65	M	G2	0	0	0	30
Itaquara	65	M	G4	0	0	0	30
Itaquara	65	F	G1	0	0	0	30
Itaquara	65	M	G4	0	0	1	30
Itaquara	66	F	G1	0	0	0	30
Itaquara	66	M	G1	0	0	0	30
Itaquara	68	M	G1	0	0	0	30
Itaquara	68	F	G1	0	0	0	30
Itaquara	68	F	G1	0	0	0	30
Itaquara	69	M	G4	0	0	2	30
Itaquara	69	M	G6	8	0	2	30
Itaquara	69	M	G1	0	0	0	30
Itaquara	70	F	G1	0	0	0	30
Itaquara	72	F	G1	0	0	0	30
Itaquara	72	F	G3	0	0	0	30
Itaquara	72	M	G1	0	0	0	30
Itaquara	73	F	G3	0	0	0	30
Itaquara	73	M	G2	0	0	0	30
Itaquara	73	F	G1	0	0	0	30

Itaquara	74	F	G1	0	0	0	30
Itaquara	76	F	G3	0	0	0	30
Itaquara	76	F	G3	0	0	0	30
Itaquara	77	F	G1	0	0	0	30
Itaquara	77	M	G3	0	0	0	30
Itaquara	79	M	G3	0	0	0	30
Itaquara	82	F	G2	0	0	1	30
Itaquara	83	M	G3	0	0	2	30
Itaquara	84	F	G1	0	0	0	30
Itaquara	85	F	G3	0	0	0	30
Itaquara	85	M	G3	0	0	0	30
Itaquara	89	F	G5	0	0	0	30
Itaquara	90	F	G1	0	0	0	30
Itaquara	93	M	G1	0	0	0	30
Itaquara	.	F	G3	0	0	0	30
Itaquara	.	M	G3	0	0	0	30
Itaquara	.	M	G1	0	0	0	30
Itaquara	.	M	G5	0	0	0	30
Cajueirinho	2	M	G4	0	0	0	30
Cajueirinho	2	F	G4	0	0	0	30
Cajueirinho	2	M	G2	0	0	0	30
Cajueirinho	3	M	G5	0	0	0	30
Cajueirinho	3	F	G5	0	0	0	30
Cajueirinho	3	F	G1	0	0	0	30
Cajueirinho	3	F	G1	0	0	0	30
Cajueirinho	3	F	G4	0	0	3	30
Cajueirinho	4	M	G5	0	0	0	30
Cajueirinho	4	M	G4	0	0	0	21
Cajueirinho	4	M	G5	0	0	0	30
Cajueirinho	4	F	G4	0	0	2	30
Cajueirinho	5	M	G1	0	0	0	30
Cajueirinho	5	M	G4	0	0	0	30
Cajueirinho	5	M	G5	0	0	0	30
Cajueirinho	5	M	G1	0	0	0	30
Cajueirinho	5	F	G6	0	0	0	30
Cajueirinho	5	M	G1	0	0	0	30
Cajueirinho	5	F	G2	0	0	0	30
Cajueirinho	5	F	G6	0	2	16	30
Cajueirinho	5	F	G4	0	0	0	30
Cajueirinho	6	M	G3	0	0	0	30
Cajueirinho	6	F	G6	0	0	0	30
Cajueirinho	6	M	G1	0	0	0	25
Cajueirinho	6	F	G3	0	0	0	30
Cajueirinho	6	M	G1	0	0	0	30
Cajueirinho	7	F	G1	0	0	0	24
Cajueirinho	7	M	G4	0	0	0	30
Cajueirinho	7	M	G5	0	0	0	30
Cajueirinho	7	F	G6	0	0	0	30
Cajueirinho	7	M	G1	0	0	0	30
Cajueirinho	7	F	G4	0	0	0	30

Cajueirinho	7	M	G1	0	0	0	30
Cajueirinho	8	F	G1	0	0	0	30
Cajueirinho	8	M	G2	0	0	1	30
Cajueirinho	8	M	G5	0	0	0	30
Cajueirinho	8	M	G3	0	0	0	30
Cajueirinho	8	F	G6	0	0	0	30
Cajueirinho	8	F	G10	0	0	0	30
Cajueirinho	8	F	G1	0	0	0	30
Cajueirinho	8	M	G6	0	0	0	30
Cajueirinho	8	M	G2	0	0	0	30
Cajueirinho	9	M	G1	0	0	0	30
Cajueirinho	9	M	G1	0	0	0	30
Cajueirinho	9	M	G5	0	0	13	30
Cajueirinho	9	M	G9	0	2	51	30
Cajueirinho	9	F	G1	0	0	0	30
Cajueirinho	10	M	G1	0	0	0	30
Cajueirinho	10	F	G4	0	0	0	26
Cajueirinho	10	M	G1	0	0	0	30
Cajueirinho	10	M	G1	0	0	0	30
Cajueirinho	10	F	G1	0	0	0	30
Cajueirinho	10	M	G4	0	0	0	30
Cajueirinho	10	M	G3	0	0	0	30
Cajueirinho	10	F	G4	0	0	6	30
Cajueirinho	11	F	G3	0	0	3	30
Cajueirinho	11	F	G4	0	0	0	30
Cajueirinho	11	F	G1	0	0	0	30
Cajueirinho	11	M	G4	0	0	0	30
Cajueirinho	11	M	G4	0	0	0	30
Cajueirinho	11	F	G4	0	0	0	30
Cajueirinho	11	M	G5	0	0	0	30
Cajueirinho	12	F	G4	0	0	0	30
Cajueirinho	12	M	G3	0	0	0	30
Cajueirinho	12	F	G3	0	0	0	30
Cajueirinho	12	F	G10	1	1	72	30
Cajueirinho	12	F	G1	0	0	0	30
Cajueirinho	12	F	G2	0	0	0	30
Cajueirinho	12	M	G1	0	0	0	30
Cajueirinho	13	M	G5	0	0	2	30
Cajueirinho	13	F	G10	0	0	0	30
Cajueirinho	13	F	G3	1	0	7	30
Cajueirinho	13	M	G1	1	0	2	30
Cajueirinho	13	M	G5	0	0	0	30
Cajueirinho	13	M	G1	0	0	0	30
Cajueirinho	13	M	G5	0	0	0	30
Cajueirinho	13	F	G5	0	0	0	30
Cajueirinho	13	M	G3	0	0	0	30
Cajueirinho	14	F	G1	0	0	0	30
Cajueirinho	14	M	G5	0	0	0	30
Cajueirinho	14	F	G5	0	0	7	30
Cajueirinho	14	F	G10	1	0	87	30

Cajueirinho	14	M	G5	0	0	0	30
Cajueirinho	14	M	G1	0	0	1	30
Cajueirinho	15	F	G4	0	0	0	30
Cajueirinho	15	M	G4	0	0	0	30
Cajueirinho	15	M	G2	0	0	5	30
Cajueirinho	15	M	G1	0	0	0	21
Cajueirinho	15	F	G5	0	0	1	30
Cajueirinho	15	F	G2	0	0	2	30
Cajueirinho	16	M	G1	0	0	0	30
Cajueirinho	16	M	G3	0	0	15	30
Cajueirinho	16	M	G1	0	0	0	30
Cajueirinho	16	M	G1	0	0	1	30
Cajueirinho	17	M	G1	0	0	0	30
Cajueirinho	17	M	G1	0	0	0	14
Cajueirinho	17	F	G2	0	0	1	30
Cajueirinho	17	F	G1	0	0	0	30
Cajueirinho	17	F	G5	0	0	2	30
Cajueirinho	17	F	G1	0	0	1	30
Cajueirinho	18	M	G2	0	0	0	30
Cajueirinho	19	F	G4	0	0	0	30
Cajueirinho	19	M	G4	0	0	0	30
Cajueirinho	19	F	G9	2	4	859	30
Cajueirinho	19	M	G2	0	0	0	30
Cajueirinho	20	F	G6	1	1	17	30
Cajueirinho	20	M	G1	1	0	2	30
Cajueirinho	20	F	G4	0	0	0	30
Cajueirinho	21	F	G3	0	0	15	30
Cajueirinho	21	F	G2	0	0	0	30
Cajueirinho	21	F	G2	0	0	0	30
Cajueirinho	21	M	G1	0	0	0	30
Cajueirinho	21	F	G3	0	0	0	30
Cajueirinho	21	F	G5	0	0	0	12
Cajueirinho	21	F	G5	2	0	1	30
Cajueirinho	22	M	G2	0	0	0	30
Cajueirinho	22	M	G4	0	0	0	30
Cajueirinho	22	F	G4	0	0	0	30
Cajueirinho	23	F	G1	0	0	0	30
Cajueirinho	23	F	G5	0	0	6	30
Cajueirinho	23	F	G7	1	0	12	30
Cajueirinho	23	F	G1	0	0	0	30
Cajueirinho	24	M	G5	0	0	3	30
Cajueirinho	24	F	G4	0	0	4	30
Cajueirinho	24	M	G3	0	0	0	30
Cajueirinho	24	F	G1	0	0	0	30
Cajueirinho	25	F	G3	0	0	0	30
Cajueirinho	25	F	G3	0	0	0	30
Cajueirinho	25	F	G1	0	0	0	30
Cajueirinho	25	F	G2	0	0	0	30
Cajueirinho	26	F	G1	0	0	0	30
Cajueirinho	26	F	G4	0	0	1	30

Cajueirinho	26	F	G1	0	0	0	30
Cajueirinho	26	F	G5	1	2	12	30
Cajueirinho	27	F	G4	0	0	0	30
Cajueirinho	27	M	G2	0	0	0	30
Cajueirinho	27	F	G4	0	0	66	11
Cajueirinho	28	M	G1	0	0	0	30
Cajueirinho	28	F	G1	0	0	1	30
Cajueirinho	28	F	G3	0	0	0	30
Cajueirinho	28	F	G10	5	6	195	30
Cajueirinho	28	F	G3	0	0	0	30
Cajueirinho	29	F	G4	0	0	0	30
Cajueirinho	29	M	G1	0	0	2	30
Cajueirinho	29	F	G1	0	0	0	30
Cajueirinho	29	F	G2	0	0	0	30
Cajueirinho	29	M	G5	0	0	3	30
Cajueirinho	29	F	G4	2	3	219	30
Cajueirinho	30	F	G4	0	0	24	30
Cajueirinho	30	M	G1	0	0	0	30
Cajueirinho	30	M	G5	0	1	0	30
Cajueirinho	30	M	G4	0	0	1	30
Cajueirinho	31	M	G4	0	0	0	14
Cajueirinho	31	M	G1	0	0	0	14
Cajueirinho	31	M	G1	0	0	0	30
Cajueirinho	32	M	G7	1	0	78	30
Cajueirinho	32	M	G1	0	0	2	30
Cajueirinho	32	F	G1	1	0	8	30
Cajueirinho	32	F	G1	0	0	0	30
Cajueirinho	33	M	G1	0	0	7	30
Cajueirinho	33	F	G3	0	0	0	30
Cajueirinho	33	F	G1	0	0	0	30
Cajueirinho	34	M	G1	0	0	0	30
Cajueirinho	34	M	G4	0	0	0	30
Cajueirinho	34	F	G1	0	0	0	30
Cajueirinho	34	M	G1	0	0	0	30
Cajueirinho	34	M	G5	1	2	1	11
Cajueirinho	34	F	G4	0	0	0	30
Cajueirinho	35	M	G1	0	0	0	30
Cajueirinho	36	F	G3	0	0	0	30
Cajueirinho	36	F	G2	0	0	0	30
Cajueirinho	36	F	G3	0	0	0	30
Cajueirinho	37	M	G6	1	0	23	30
Cajueirinho	37	F	G7	2	4	4	30
Cajueirinho	37	F	G2	0	0	0	30
Cajueirinho	37	F	G4	0	0	0	30
Cajueirinho	37	M	G1	0	0	0	30
Cajueirinho	38	F	G10	0	0	2	30
Cajueirinho	38	M	G3	0	0	0	30
Cajueirinho	38	M	G4	0	0	2	30
Cajueirinho	39	M	G2	0	0	0	30
Cajueirinho	39	F	G1	0	0	1	30

Cajueirinho	40	F	G1	1	0	3	30
Cajueirinho	40	M	G1	0	0	4	30
Cajueirinho	41	F	G1	0	0	0	30
Cajueirinho	42	M	G2	0	1	5	30
Cajueirinho	43	F	G1	0	0	0	30
Cajueirinho	44	F	G1	0	0	0	30
Cajueirinho	44	M	G1	1	0	1	30
Cajueirinho	44	F	G1	2	0	1	30
Cajueirinho	45	M	G1	0	0	0	30
Cajueirinho	45	F	G5	0	0	1	30
Cajueirinho	45	M	G5	1	0	6	30
Cajueirinho	45	F	G1	0	0	0	30
Cajueirinho	45	F	G1	0	0	0	30
Cajueirinho	46	M	G1	0	0	0	30
Cajueirinho	46	F	G3	0	0	0	30
Cajueirinho	47	M	G3	0	0	0	30
Cajueirinho	47	F	G1	0	0	0	30
Cajueirinho	47	M	G1	0	0	0	30
Cajueirinho	47	M	G1	0	0	1	30
Cajueirinho	47	F	G1	0	0	1	30
Cajueirinho	48	F	G8	5	4	22	30
Cajueirinho	48	F	G1	0	0	0	30
Cajueirinho	48	F	G4	0	0	1	30
Cajueirinho	49	M	G3	0	0	0	30
Cajueirinho	49	F	G4	0	0	0	30
Cajueirinho	49	F	G1	0	0	0	30
Cajueirinho	49	F	G1	0	0	0	30
Cajueirinho	50	M	G1	0	0	1	30
Cajueirinho	50	M	G1	0	0	3	30
Cajueirinho	50	M	G1	0	0	0	30
Cajueirinho	50	M	G4	0	0	0	30
Cajueirinho	51	M	G3	0	0	0	30
Cajueirinho	51	F	G1	0	0	14	30
Cajueirinho	52	M	G1	0	0	0	30
Cajueirinho	52	M	G1	0	0	1	30
Cajueirinho	52	F	G4	1	3	10	24
Cajueirinho	52	F	G1	0	0	0	30
Cajueirinho	52	F	G1	0	0	0	30
Cajueirinho	53	M	G4	0	0	0	21
Cajueirinho	53	M	G1	0	0	0	30
Cajueirinho	53	M	G6	0	0	9	30
Cajueirinho	53	F	G4	1	0	6	30
Cajueirinho	53	M	G5	0	0	0	30
Cajueirinho	53	M	G2	0	0	0	30
Cajueirinho	54	F	G1	1	0	11	30
Cajueirinho	54	M	G1	0	0	20	30
Cajueirinho	55	F	G2	0	0	0	30
Cajueirinho	55	M	G4	0	0	5	30
Cajueirinho	55	M	G2	0	0	0	30
Cajueirinho	56	F	G1	0	0	0	30

Cajueirinho	57	M	G1	0	0	1	30
Cajueirinho	57	M	G1	0	0	0	30
Cajueirinho	58	F	G2	0	0	0	30
Cajueirinho	59	F	G2	0	0	3	30
Cajueirinho	60	M	G5	3	2	3	30
Cajueirinho	60	M	G3	0	0	0	13
Cajueirinho	64	F	G1	0	0	0	30
Cajueirinho	64	M	G1	0	0	0	30
Cajueirinho	64	M	G1	0	0	0	30
Cajueirinho	65	M	G3	0	0	0	30
Cajueirinho	65	M	G3	0	0	0	30
Cajueirinho	66	F	G1	0	0	0	30
Cajueirinho	67	F	G4	0	0	0	30
Cajueirinho	67	M	G1	0	0	0	30
Cajueirinho	67	M	G1	1	0	0	30
Cajueirinho	68	F	G1	0	0	0	30
Cajueirinho	69	M	G5	0	0	66	30
Cajueirinho	71	F	G1	0	0	0	30
Cajueirinho	73	M	G1	0	0	0	30
Cajueirinho	78	F	G3	0	0	8	30
Cajueirinho	78	F	G4	0	0	5	30
Cajueirinho	82	F	G2	0	0	0	30
Cajueirinho	87	M	G6	0	0	0	30
Jaguaritira	6	F	G4	0	0	0	32
Jaguaritira	6	M	G5	0	0	0	30
Jaguaritira	6	F	G6	0	0	0	30
Jaguaritira	6	M	G4	0	0	18	30
Jaguaritira	6	F	G5	0	0	168	30
Jaguaritira	6	M	G1	0	0	3	30
Jaguaritira	6	M	G5	0	0	1	23
Jaguaritira	7	F	G5	0	0	0	30
Jaguaritira	7	M	G4	0	0	205	30
Jaguaritira	7	M	G5	0	0	5	30
Jaguaritira	7	M	G5	0	0	0	23
Jaguaritira	7	M	G3	0	0	0	30
Jaguaritira	7	F	G3	0	0	2	30
Jaguaritira	8	M	G3	0	0	0	30
Jaguaritira	8	M	G4	0	0	0	30
Jaguaritira	8	F	G4	0	0	0	30
Jaguaritira	8	M	G3	0	0	2	30
Jaguaritira	8	F	G4	0	0	1	30
Jaguaritira	8	M	G8	4	1	12	23
Jaguaritira	9	F	G1	0	0	2	28
Jaguaritira	9	M	G7	0	0	0	30
Jaguaritira	9	M	G9	5	4	525	16
Jaguaritira	9	M	G3	0	0	0	30
Jaguaritira	10	F	G4	0	0	1	30
Jaguaritira	10	M	G4	0	0	0	30
Jaguaritira	10	F	G5	0	0	0	30
Jaguaritira	10	M	G5	0	0	1	30

Jaguaritira	10	M	G5	0	0	12	30
Jaguaritira	10	F	G9	6	4	68	30
Jaguaritira	10	M	G5	2	1	5	30
Jaguaritira	10	F	G4	0	0	2	30
Jaguaritira	10	F	G8	0	0	12	30
Jaguaritira	10	M	G6	0	0	0	30
Jaguaritira	10	M	G4	0	0	16	16
Jaguaritira	11	M	G5	6	7	30	30
Jaguaritira	11	M	G1	0	0	0	30
Jaguaritira	11	M	G7	0	1	94	30
Jaguaritira	11	F	G1	0	0	181	30
Jaguaritira	11	F	G10	56	68	1000	25
Jaguaritira	11	M	G9	2	0	64	30
Jaguaritira	11	F	G5	0	0	4	30
Jaguaritira	11	F	G1	0	0	3	30
Jaguaritira	11	F	G10	66	43	276	18
Jaguaritira	11	F	G6	0	1	39	30
Jaguaritira	11	M	G6	1	0	5	30
Jaguaritira	11	M	G7	0	0	1	30
Jaguaritira	12	F	G7	1	0	50	30
Jaguaritira	12	M	G4	4	3	89	30
Jaguaritira	12	M	G4	0	0	0	31
Jaguaritira	12	F	G5	0	0	0	30
Jaguaritira	12	M	G5	0	0	0	30
Jaguaritira	12	F	G5	0	0	0	30
Jaguaritira	12	M	G2	0	0	0	31
Jaguaritira	12	F	G4	0	0	0	30
Jaguaritira	12	F	G4	0	0	0	27
Jaguaritira	12	M	G6	1	5	5	30
Jaguaritira	12	M	G5	0	0	0	30
Jaguaritira	12	M	G5	0	0	4	30
Jaguaritira	12	F	G10	49	43	2608	30
Jaguaritira	12	M	G5	0	0	1	28
Jaguaritira	12	F	G4	0	0	0	29
Jaguaritira	12	M	G9	19	23	808	30
Jaguaritira	12	M	G5	0	0	2	31
Jaguaritira	12	F	G8	0	0	39	30
Jaguaritira	12	F	G1	0	0	1	2
Jaguaritira	12	M	G1	0	0	0	30
Jaguaritira	13	M	G3	0	0	0	30
Jaguaritira	13	M	G3	0	0	2	30
Jaguaritira	13	F	G4	0	0	0	30
Jaguaritira	13	F	G2	0	0	0	30
Jaguaritira	13	M	G1	0	0	0	30
Jaguaritira	13	M	G3	0	0	9	30
Jaguaritira	13	F	G7	0	0	0	30
Jaguaritira	13	M	G6	0	0	10	30
Jaguaritira	13	M	G3	0	0	3	30
Jaguaritira	13	F	G5	0	0	16	30
Jaguaritira	13	F	G5	0	0	0	30

Jaguaritira	13	F	G9	1	1	75	30
Jaguaritira	13	M	G1	0	0	0	30
Jaguaritira	14	F	G3	0	0	0	30
Jaguaritira	14	M	G10	7	5	1	30
Jaguaritira	14	M	G6	0	0	54	30
Jaguaritira	14	M	G7	0	0	20	19
Jaguaritira	14	M	G4	0	0	1	30
Jaguaritira	14	M	G10	45	33	3216	30
Jaguaritira	14	M	G8	73	68	230	30
Jaguaritira	14	F	G6	0	0	10	27
Jaguaritira	14	F	G5	0	0	4	30
Jaguaritira	14	M	G4	0	0	0	30
Jaguaritira	14	F	G5	0	0	5	30
Jaguaritira	15	M	G10	0	0	41	30
Jaguaritira	15	F	G5	0	0	27	28
Jaguaritira	15	F	G1	0	0	0	30
Jaguaritira	15	F	G7	0	0	1	30
Jaguaritira	15	F	G3	0	0	19	30
Jaguaritira	15	F	G4	0	0	0	30
Jaguaritira	15	M	G1	0	0	0	27
Jaguaritira	15	M	G6	1	0	29	30
Jaguaritira	15	M	G1	0	0	0	24
Jaguaritira	16	M	G6	0	0	6	30
Jaguaritira	16	F	G3	0	0	0	30
Jaguaritira	16	M	G6	4	3	120	30
Jaguaritira	16	F	G7	0	0	30	30
Jaguaritira	16	F	G7	0	0	3	30
Jaguaritira	17	F	G4	0	0	1	30
Jaguaritira	17	M	G1	0	0	0	30
Jaguaritira	17	M	G4	0	0	3	30
Jaguaritira	17	F	G1	0	0	0	30
Jaguaritira	17	M	G3	0	0	3	30
Jaguaritira	17	F	G2	0	0	0	30
Jaguaritira	18	F	G3	0	3	6	30
Jaguaritira	18	M	G5	0	0	0	30
Jaguaritira	18	M	G1	0	0	0	27
Jaguaritira	19	M	G6	3	0	331	30
Jaguaritira	19	M	G1	0	0	0	30
Jaguaritira	20	M	G1	2	1	0	30
Jaguaritira	20	M	G7	0	0	1	28
Jaguaritira	22	F	G7	0	0	0	30
Jaguaritira	22	F	G1	0	0	1	30
Jaguaritira	24	F	G6	3	2	68	30
Jaguaritira	25	M	G3	0	0	2	30
Jaguaritira	25	M	G9	17	2	0	30
Jaguaritira	26	F	G5	0	0	0	30
Jaguaritira	27	F	G5	0	0	0	30
Jaguaritira	27	M	G5	1	0	0	30
Jaguaritira	27	F	G3	2	0	0	30
Jaguaritira	28	F	G4	0	0	0	30

Jaguaritira	29	F	G8	9	10	0	30
Jaguaritira	29	F	G5	0	0	1	30
Jaguaritira	30	F	G4	0	0	5	30
Jaguaritira	30	F	G3	0	0	1	30
Jaguaritira	30	F	G3	0	0	0	30
Jaguaritira	30	M	G1	0	0	0	27
Jaguaritira	31	F	G4	0	0	12	30
Jaguaritira	31	F	G5	0	0	2	32
Jaguaritira	31	F	G5	0	0	3	30
Jaguaritira	32	F	G9	32	21	601	20
Jaguaritira	32	F	G5	0	0	48	29
Jaguaritira	32	F	G3	0	0	3	32
Jaguaritira	32	F	G5	0	0	0	30
Jaguaritira	32	F	G3	0	0	0	30
Jaguaritira	32	M	G2	0	0	3	30
Jaguaritira	32	F	G5	0	0	2	21
Jaguaritira	32	M	G1	0	0	0	30
Jaguaritira	32	F	G5	0	0	0	30
Jaguaritira	32	F	G4	0	0	11	30
Jaguaritira	33	M	G2	0	0	7	26
Jaguaritira	33	M	G1	0	0	0	30
Jaguaritira	33	F	G1	0	0	0	30
Jaguaritira	33	F	G1	0	0	4	30
Jaguaritira	33	F	G4	0	0	0	30
Jaguaritira	34	F	G6	1	3	12	31
Jaguaritira	34	M	G2	0	0	0	30
Jaguaritira	34	M	G4	0	0	1	30
Jaguaritira	34	F	G3	0	0	0	30
Jaguaritira	34	F	G7	3	6	35	30
Jaguaritira	34	F	G4	0	0	0	32
Jaguaritira	35	M	G1	0	0	3	30
Jaguaritira	35	M	G3	0	0	0	30
Jaguaritira	35	F	G5	0	0	0	30
Jaguaritira	36	F	G4	0	0	0	30
Jaguaritira	36	M	G2	0	0	10	28
Jaguaritira	36	F	G1	0	0	0	30
Jaguaritira	36	F	G5	0	0	0	26
Jaguaritira	36	M	G6	0	0	4	30
Jaguaritira	37	F	G1	0	0	1	30
Jaguaritira	37	F	G5	20	11	895	30
Jaguaritira	37	M	G1	0	0	0	30
Jaguaritira	37	F	G2	0	0	3	31
Jaguaritira	37	M	G4	1	0	18	17
Jaguaritira	37	M	G1	0	0	0	30
Jaguaritira	38	F	G2	0	0	2	30
Jaguaritira	38	M	G5	1	1	64	30
Jaguaritira	38	M	G3	0	0	0	30
Jaguaritira	38	F	G5	0	0	1	30
Jaguaritira	38	M	G3	0	0	0	30
Jaguaritira	39	M	G7	2	5	260	30

Jaguaritira	39	F	G1	2	0	23	30
Jaguaritira	39	M	G3	0	0	0	30
Jaguaritira	39	F	G5	0	0	0	30
Jaguaritira	39	F	G1	0	0	0	30
Jaguaritira	39	F	G9	7	10	682	30
Jaguaritira	39	F	G4	0	0	4	30
Jaguaritira	40	F	G3	0	0	3	30
Jaguaritira	40	M	G1	0	0	1	30
Jaguaritira	40	M	G3	1	0	0	30
Jaguaritira	41	F	G1	0	0	0	29
Jaguaritira	41	M	G3	0	0	4	30
Jaguaritira	41	M	G1	0	0	31	30
Jaguaritira	42	M	G2	0	0	0	27
Jaguaritira	42	M	G2	0	0	19	30
Jaguaritira	42	M	G1	0	0	3	30
Jaguaritira	42	F	G6	0	0	326	30
Jaguaritira	43	M	G4	0	0	0	30
Jaguaritira	43	M	G6	0	0	0	30
Jaguaritira	43	F	G3	0	0	0	30
Jaguaritira	43	F	G1	0	0	8	31
Jaguaritira	43	F	G1	0	0	0	30
Jaguaritira	44	F	G2	0	0	0	30
Jaguaritira	44	M	G8	1	0	11	30
Jaguaritira	44	M	G2	0	1	1	21
Jaguaritira	45	F	G7	1	0	89	30
Jaguaritira	45	M	G5	0	0	0	29
Jaguaritira	45	M	G4	0	0	1	30
Jaguaritira	46	M	G3	0	0	0	30
Jaguaritira	46	F	G5	0	0	11	30
Jaguaritira	47	M	G6	0	0	1	30
Jaguaritira	47	F	G1	0	0	0	30
Jaguaritira	47	M	G1	0	0	0	30
Jaguaritira	47	F	G5	0	0	0	30
Jaguaritira	47	F	G9	4	8	396	30
Jaguaritira	48	F	G5	0	0	0	16
Jaguaritira	48	M	G1	0	0	0	30
Jaguaritira	48	F	G1	0	0	0	30
Jaguaritira	49	F	G3	0	0	0	31
Jaguaritira	50	F	G1	0	0	2	30
Jaguaritira	50	F	G3	0	0	0	30
Jaguaritira	50	M	G1	0	0	3	31
Jaguaritira	51	M	G2	0	0	2	30
Jaguaritira	51	F	G1	0	0	3	30
Jaguaritira	51	F	G1	0	0	0	31
Jaguaritira	52	F	G1	4	0	5	30
Jaguaritira	53	F	G1	0	0	0	30
Jaguaritira	53	F	G1	0	0	0	30
Jaguaritira	54	M	G2	0	0	0	30
Jaguaritira	54	F	G1	0	0	111	30
Jaguaritira	55	F	G1	0	0	3	30

Jaguaritira	55	F	G1	0	0	0	8
Jaguaritira	57	M	G7	1	5	70	29
Jaguaritira	57	M	G4	0	0	2	30
Jaguaritira	57	M	G1	0	0	0	30
Jaguaritira	58	M	G7	4	3	73	30
Jaguaritira	58	F	G6	0	0	0	30
Jaguaritira	59	M	G1	0	0	2	30
Jaguaritira	59	M	G4	0	0	6	30
Jaguaritira	60	M	G3	0	0	0	30
Jaguaritira	60	M	G5	0	2	1	30
Jaguaritira	61	F	G1	0	0	0	29
Jaguaritira	64	M	G1	0	0	0	30
Jaguaritira	65	F	G1	0	0	2	30
Jaguaritira	72	M	G5	5	1	1	30
Jaguaritira	74	M	G5	0	0	0	30
Sempre Viva	4	F	G5	0	0	0	9
Sempre Viva	5	F	G3	0	0	1	30
Sempre Viva	5	F	G1	0	0	0	30
Sempre Viva	5	F	G4	0	0	1	30
Sempre Viva	5	F	G4	0	0	5	30
Sempre Viva	5	M	G1	0	0	0	26
Sempre Viva	5	F	G7	0	0	3	30
Sempre Viva	5	F	G3	0	0	0	30
Sempre Viva	5	M	G4	0	0	2	30
Sempre Viva	5	M	G1	0	0	0	30
Sempre Viva	6	F	G3	0	0	0	30
Sempre Viva	6	F	G3	0	0	1	30
Sempre Viva	6	M	G2	0	0	0	29
Sempre Viva	6	M	G4	0	0	0	28
Sempre Viva	6	M	G4	0	0	0	30
Sempre Viva	6	M	G2	0	0	0	30
Sempre Viva	6	M	G5	0	0	0	30
Sempre Viva	6	M	G8	0	0	1	30
Sempre Viva	6	M	G9	0	0	0	30
Sempre Viva	6	M	G2	0	0	0	30
Sempre Viva	7	F	G1	0	0	0	27
Sempre Viva	7	M	G4	0	0	0	30
Sempre Viva	7	M	G4	1	0	0	30
Sempre Viva	7	F	G1	0	0	0	30
Sempre Viva	7	M	G4	0	0	0	30
Sempre Viva	7	F	G4	0	0	0	30
Sempre Viva	7	M	G3	1	0	0	29
Sempre Viva	7	F	G3	0	0	0	30
Sempre Viva	7	M	G2	0	0	0	30
Sempre Viva	7	F	G2	0	0	0	30
Sempre Viva	8	F	G1	0	0	6	30
Sempre Viva	8	F	G10	0	0	10	30
Sempre Viva	8	F	G8	7	0	21	30
Sempre Viva	8	M	G6	0	0	0	30
Sempre Viva	8	F	G3	0	0	0	30

Sempre Viva	8	M	G3	0	0	0	30
Sempre Viva	8	M	G2	0	0	0	30
Sempre Viva	8	M	G1	0	0	0	30
Sempre Viva	8	M	G7	0	1	1	30
Sempre Viva	8	F	G1	0	0	0	23
Sempre Viva	9	F	G5	0	0	7	13
Sempre Viva	9	F	G8	0	0	1	30
Sempre Viva	9	M	G6	0	0	0	30
Sempre Viva	9	M	G5	0	0	0	30
Sempre Viva	9	F	G9	0	1	22	30
Sempre Viva	9	F	G3	0	0	0	30
Sempre Viva	9	M	G2	0	0	0	30
Sempre Viva	9	M	G7	0	0	2	30
Sempre Viva	9	M	G5	0	0	64	30
Sempre Viva	9	F	G6	0	0	0	30
Sempre Viva	9	M	G8	1	0	25	30
Sempre Viva	9	F	G4	0	0	0	30
Sempre Viva	9	M	G4	0	0	0	23
Sempre Viva	9	M	G1	0	0	0	16
Sempre Viva	10	F	G3	0	0	2	20
Sempre Viva	10	M	G7	0	1	3	30
Sempre Viva	10	F	G6	0	0	35	30
Sempre Viva	10	F	G7	0	0	0	30
Sempre Viva	10	M	G2	0	0	0	19
Sempre Viva	10	M	G1	0	0	0	30
Sempre Viva	11	M	G1	0	0	0	30
Sempre Viva	11	F	G4	0	0	0	30
Sempre Viva	11	M	G6	0	0	0	30
Sempre Viva	11	M	G4	1	3	40	15
Sempre Viva	11	F	G7	0	0	0	25
Sempre Viva	11	F	G4	0	0	0	11
Sempre Viva	11	M	G8	0	0	1	11
Sempre Viva	11	M	G5	0	0	0	8
Sempre Viva	11	F	G6	0	1	0	28
Sempre Viva	11	F	G5	0	0	1	30
Sempre Viva	11	M	G2	0	0	0	11
Sempre Viva	11	F	G4	0	0	0	12
Sempre Viva	11	F	G6	0	0	0	30
Sempre Viva	12	M	G3	0	0	0	30
Sempre Viva	12	F	G3	15	19	31	14
Sempre Viva	12	F	G4	0	0	0	30
Sempre Viva	12	M	G1	0	0	0	30
Sempre Viva	12	M	G7	0	0	0	30
Sempre Viva	12	F	G8	0	0	10	30
Sempre Viva	12	M	G8	5	4	37	30
Sempre Viva	12	F	G4	0	0	0	30
Sempre Viva	12	M	G1	0	0	16	30
Sempre Viva	13	F	G9	6	11	23	25
Sempre Viva	13	F	G7	0	0	0	30
Sempre Viva	13	F	G1	0	0	0	30

Sempre Viva	13	M	G9	1	1	0	30
Sempre Viva	13	M	G10	1	0	11	25
Sempre Viva	13	M	G3	0	0	1	30
Sempre Viva	13	M	G3	0	0	0	30
Sempre Viva	14	F	G10	2	1	4	30
Sempre Viva	14	M	G8	0	0	0	30
Sempre Viva	14	F	G5	7	16	0	30
Sempre Viva	14	M	G9	5	3	8	30
Sempre Viva	14	M	G8	2	11	4	8
Sempre Viva	14	F	G1	0	0	0	30
Sempre Viva	14	M	G9	1	2	6	15
Sempre Viva	14	F	G2	0	0	0	30
Sempre Viva	14	M	G2	0	0	0	30
Sempre Viva	14	F	G1	0	0	0	10
Sempre Viva	15	M	G7	2	3	27	20
Sempre Viva	15	M	G8	4	0	2	30
Sempre Viva	15	F	G5	0	0	0	28
Sempre Viva	15	F	G9	1	2	1	30
Sempre Viva	15	F	G3	0	1	228	30
Sempre Viva	15	F	G10	0	0	6	30
Sempre Viva	15	M	G4	0	0	0	17
Sempre Viva	15	F	G1	0	0	0	12
Sempre Viva	16	F	G4	5	5	46	15
Sempre Viva	16	F	G9	3	2	57	30
Sempre Viva	16	M	G6	7	6	9	30
Sempre Viva	16	F	G1	0	0	4	30
Sempre Viva	16	F	G7	0	0	0	30
Sempre Viva	16	M	G6	0	0	0	30
Sempre Viva	17	F	G1	0	0	0	30
Sempre Viva	17	F	G9	5	13	5	30
Sempre Viva	17	M	G10	4	4	2	30
Sempre Viva	17	M	G6	0	0	0	26
Sempre Viva	18	F	G4	0	0	16	10
Sempre Viva	18	F	G7	2	6	4	30
Sempre Viva	18	M	G6	2	0	0	14
Sempre Viva	18	F	G2	0	0	0	19
Sempre Viva	18	F	G3	0	0	0	23
Sempre Viva	19	F	G1	0	0	2	30
Sempre Viva	19	M	G1	0	0	1	30
Sempre Viva	19	M	G1	18	8	3	30
Sempre Viva	19	F	G10	0	1	189	30
Sempre Viva	19	F	G4	0	1	0	30
Sempre Viva	20	F	G3	0	0	0	18
Sempre Viva	20	M	G7	4	2	51	30
Sempre Viva	20	F	G7	0	1	7	30
Sempre Viva	20	F	G10	0	0	1	30
Sempre Viva	20	M	G1	0	0	0	30
Sempre Viva	21	M	G9	31	21	453	30
Sempre Viva	21	F	G2	0	0	0	30
Sempre Viva	21	F	G3	0	0	0	30

Sempre Viva	21	F	G3	0	0	0	8
Sempre Viva	22	M	G3	0	0	0	30
Sempre Viva	22	M	G9	1	3	0	30
Sempre Viva	22	F	G1	1	0	0	30
Sempre Viva	22	F	G1	0	0	0	4
Sempre Viva	23	F	G9	1	3	2	30
Sempre Viva	23	F	G10	2	0	0	30
Sempre Viva	23	F	G5	1	1	72	30
Sempre Viva	23	F	G4	0	0	0	6
Sempre Viva	23	F	G2	0	0	0	30
Sempre Viva	23	F	G3	0	0	0	23
Sempre Viva	23	F	G1	0	0	0	20
Sempre Viva	23	F	G1	0	0	0	16
Sempre Viva	24	F	G6	4	1	12	30
Sempre Viva	24	F	G8	3	10	1	30
Sempre Viva	24	F	G3	0	2	1	26
Sempre Viva	24	F	G3	0	0	0	30
Sempre Viva	24	F	G8	0	0	0	30
Sempre Viva	24	F	G10	1	1	4	30
Sempre Viva	24	M	G2	0	0	0	12
Sempre Viva	24	M	G6	9	8	48	30
Sempre Viva	24	F	G1	0	0	0	30
Sempre Viva	24	M	G1	0	0	0	30
Sempre Viva	25	M	G1	2	0	23	30
Sempre Viva	25	M	G5	4	3	18	30
Sempre Viva	25	M	G1	0	0	0	30
Sempre Viva	25	F	G8	0	1	3	30
Sempre Viva	25	F	G6	0	0	0	30
Sempre Viva	25	M	G3	0	4	0	30
Sempre Viva	25	M	G4	0	0	0	30
Sempre Viva	26	F	G8	1	3	8	30
Sempre Viva	26	M	G1	0	0	0	30
Sempre Viva	26	F	G5	2	2	1	30
Sempre Viva	26	M	G2	0	0	0	30
Sempre Viva	26	F	G2	0	0	0	9
Sempre Viva	27	M	G7	9	8	1	26
Sempre Viva	27	F	G1	0	0	6	30
Sempre Viva	27	F	G4	0	0	0	30
Sempre Viva	27	F	G2	0	0	3	30
Sempre Viva	27	M	G10	10	13	51	30
Sempre Viva	27	F	G6	0	0	0	30
Sempre Viva	27	F	G1	0	0	0	30
Sempre Viva	27	M	G5	3	3	31	30
Sempre Viva	27	F	G6	0	0	0	30
Sempre Viva	27	F	G1	0	0	0	30
Sempre Viva	27	M	G1	0	0	0	30
Sempre Viva	27	F	G1	0	0	0	30
Sempre Viva	28	M	G4	0	0	6	30
Sempre Viva	28	F	G10	2	4	1	30
Sempre Viva	28	F	G6	0	0	4	30

Sempre Viva	28	F	G1	0	0	0	30
Sempre Viva	28	F	G6	5	3	245	30
Sempre Viva	28	F	G8	0	0	7	30
Sempre Viva	28	M	G2	0	0	0	30
Sempre Viva	28	F	G4	0	0	0	30
Sempre Viva	29	F	G3	0	0	5	30
Sempre Viva	29	F	G1	0	0	1	30
Sempre Viva	29	F	G6	0	0	0	30
Sempre Viva	29	F	G2	0	0	0	30
Sempre Viva	29	M	G1	0	0	0	15
Sempre Viva	30	F	G1	2	0	2	30
Sempre Viva	30	F	G2	2	0	0	30
Sempre Viva	30	M	G1	1	0	2	30
Sempre Viva	31	F	G1	0	0	1	30
Sempre Viva	31	F	G1	0	0	1	30
Sempre Viva	31	F	G1	1	1	2	11
Sempre Viva	31	F	G3	0	0	0	30
Sempre Viva	32	F	G4	0	0	3	30
Sempre Viva	32	M	G1	0	0	0	30
Sempre Viva	32	F	G3	1	0	0	30
Sempre Viva	32	F	G3	0	0	3	30
Sempre Viva	33	F	G1	0	0	3	30
Sempre Viva	33	M	G3	0	0	1	30
Sempre Viva	33	F	G2	0	0	0	12
Sempre Viva	33	F	G5	0	0	0	11
Sempre Viva	34	F	G7	1	0	33	30
Sempre Viva	34	M	G1	0	1	0	30
Sempre Viva	34	F	G3	0	0	0	30
Sempre Viva	34	F	G3	1	1	8	14
Sempre Viva	34	F	G1	0	0	0	30
Sempre Viva	34	F	G10	23	27	9	30
Sempre Viva	34	F	G1	0	0	1	30
Sempre Viva	34	M	G1	0	0	0	26
Sempre Viva	34	F	G1	0	0	0	20
Sempre Viva	35	F	G2	2	0	4	30
Sempre Viva	35	M	G6	14	17	16	30
Sempre Viva	35	M	G1	0	0	0	30
Sempre Viva	35	F	G1	0	0	0	20
Sempre Viva	36	M	G8	11	10	0	30
Sempre Viva	37	F	G1	0	0	0	30
Sempre Viva	37	F	G7	0	0	0	30
Sempre Viva	37	M	G9	2	6	154	30
Sempre Viva	37	M	G1	0	0	0	30
Sempre Viva	37	F	G1	0	0	0	30
Sempre Viva	37	M	G2	0	0	3	30
Sempre Viva	37	F	G5	0	0	0	30
Sempre Viva	38	F	G1	0	0	0	30
Sempre Viva	38	M	G8	0	1	16	30
Sempre Viva	38	M	G2	0	1	1	30
Sempre Viva	38	F	G4	0	0	0	10

Sempre Viva	38	M	G1	0	0	0	30
Sempre Viva	39	M	G1	0	0	0	12
Sempre Viva	39	M	G1	0	0	2	30
Sempre Viva	39	M	G8	4	1	1	30
Sempre Viva	39	M	G5	0	1	0	30
Sempre Viva	39	F	G1	0	0	0	10
Sempre Viva	39	M	G1	0	0	0	17
Sempre Viva	40	M	G4	0	0	0	30
Sempre Viva	40	F	G1	0	0	0	30
Sempre Viva	40	F	G3	0	0	0	30
Sempre Viva	41	M	G1	0	0	0	30
Sempre Viva	41	M	G1	1	7	6	30
Sempre Viva	41	F	G3	2	0	0	30
Sempre Viva	41	F	G1	0	0	0	30
Sempre Viva	41	F	G1	0	0	0	30
Sempre Viva	41	M	G1	0	0	0	30
Sempre Viva	42	M	G1	0	0	3	30
Sempre Viva	42	M	G1	1	0	14	30
Sempre Viva	42	F	G3	2	0	51	30
Sempre Viva	42	F	G4	0	0	0	30
Sempre Viva	43	M	G3	0	0	0	30
Sempre Viva	43	F	G2	0	0	0	30
Sempre Viva	43	F	G5	0	0	0	30
Sempre Viva	43	M	G3	1	1	4	30
Sempre Viva	43	M	G4	3	0	0	30
Sempre Viva	43	F	G1	0	0	0	18
Sempre Viva	43	F	G4	0	0	1	30
Sempre Viva	44	F	G2	0	0	0	30
Sempre Viva	44	F	G2	0	0	3	30
Sempre Viva	44	M	G5	1	0	16	22
Sempre Viva	44	F	G4	0	0	0	30
Sempre Viva	44	F	G1	0	0	0	30
Sempre Viva	45	F	G4	0	0	0	30
Sempre Viva	45	F	G2	0	0	0	30
Sempre Viva	45	M	G3	3	1	1	6
Sempre Viva	45	F	G1	0	0	0	30
Sempre Viva	47	M	G8	2	4	29	30
Sempre Viva	47	F	G1	0	0	0	30
Sempre Viva	47	F	G2	0	0	3	30
Sempre Viva	47	F	G2	0	0	0	30
Sempre Viva	47	M	G1	0	0	0	5
Sempre Viva	47	F	G1	0	0	0	30
Sempre Viva	47	F	G1	0	0	0	6
Sempre Viva	47	F	G4	0	0	0	25
Sempre Viva	47	F	G4	0	0	0	30
Sempre Viva	48	F	G3	0	0	0	30
Sempre Viva	48	M	G5	4	2	98	30
Sempre Viva	48	F	G6	1	0	0	30
Sempre Viva	48	M	G5	0	0	0	30
Sempre Viva	49	F	G1	0	0	0	30

Sempre Viva	49	F	G1	0	0	0	30
Sempre Viva	49	F	G1	0	0	0	30
Sempre Viva	49	M	G1	0	0	0	30
Sempre Viva	49	M	G3	0	0	0	30
Sempre Viva	49	F	G7	0	0	26	30
Sempre Viva	49	F	G4	2	3	0	30
Sempre Viva	49	M	G10	6	6	12	5
Sempre Viva	49	F	G1	0	0	0	30
Sempre Viva	49	F	G2	0	0	1	30
Sempre Viva	49	M	G1	0	0	0	28
Sempre Viva	49	M	G1	0	0	0	30
Sempre Viva	50	M	G1	0	0	0	30
Sempre Viva	50	M	G7	1	1	0	30
Sempre Viva	50	F	G2	1	0	7	30
Sempre Viva	50	F	G1	0	0	0	30
Sempre Viva	50	F	G1	0	0	0	30
Sempre Viva	50	F	G1	0	0	0	12
Sempre Viva	51	M	G6	1	0	2	30
Sempre Viva	51	F	G2	0	0	0	30
Sempre Viva	51	F	G1	0	0	1	30
Sempre Viva	51	F	G4	0	0	0	30
Sempre Viva	52	F	G2	0	0	0	30
Sempre Viva	52	F	G1	0	0	0	30
Sempre Viva	52	F	G1	0	0	0	30
Sempre Viva	52	F	G3	0	0	0	30
Sempre Viva	52	F	G1	0	0	0	30
Sempre Viva	52	M	G3	0	0	0	19
Sempre Viva	52	M	G4	0	0	0	30
Sempre Viva	53	F	G4	0	0	0	30
Sempre Viva	53	F	G1	0	1	0	30
Sempre Viva	53	M	G3	0	0	4	19
Sempre Viva	53	F	G1	0	0	0	30
Sempre Viva	53	M	G9	53	53	2638	30
Sempre Viva	53	F	G8	3	11	2	30
Sempre Viva	53	M	G4	0	0	0	30
Sempre Viva	53	F	G1	0	0	0	30
Sempre Viva	54	F	G1	0	0	0	30
Sempre Viva	54	F	G4	0	0	0	30
Sempre Viva	54	F	G1	0	0	0	30
Sempre Viva	54	F	G2	0	0	0	30
Sempre Viva	54	F	G5	0	0	0	30
Sempre Viva	54	F	G2	0	0	0	30
Sempre Viva	55	M	G5	0	1	36	30
Sempre Viva	55	F	G3	10	22	181	30
Sempre Viva	55	F	G2	0	0	0	30
Sempre Viva	55	M	G5	0	2	0	30
Sempre Viva	55	F	G3	0	0	0	30
Sempre Viva	55	F	G1	0	0	0	30
Sempre Viva	55	M	G2	0	0	0	30
Sempre Viva	55	M	G1	0	0	0	4

Sempre Viva	56	M	G1	0	0	0	30
Sempre Viva	56	F	G4	0	0	0	30
Sempre Viva	56	F	G1	0	0	0	30
Sempre Viva	56	F	G1	0	0	0	30
Sempre Viva	57	F	G1	0	0	0	30
Sempre Viva	57	M	G2	0	0	0	30
Sempre Viva	57	F	G1	0	0	3	30
Sempre Viva	57	F	G4	0	0	0	30
Sempre Viva	57	M	G1	0	0	0	30
Sempre Viva	57	M	G1	0	0	0	15
Sempre Viva	57	F	G1	0	0	0	30
Sempre Viva	58	F	G1	0	0	7	30
Sempre Viva	58	M	G9	41	37	383	30
Sempre Viva	58	M	G4	0	0	0	30
Sempre Viva	58	M	G1	1	0	0	30
Sempre Viva	59	M	G10	7	8	37	30
Sempre Viva	59	F	G1	0	0	0	30
Sempre Viva	59	F	G1	0	0	0	30
Sempre Viva	60	M	G1	0	0	0	30
Sempre Viva	60	F	G1	0	0	0	16
Sempre Viva	62	F	G1	0	0	5	30
Sempre Viva	62	F	G1	0	0	5	30
Sempre Viva	62	F	G3	0	0	0	11
Sempre Viva	63	F	G7	7	7	101	24
Sempre Viva	63	F	G10	6	6	194	30
Sempre Viva	63	M	G4	0	0	0	30
Sempre Viva	64	M	G10	1	4	96	23
Sempre Viva	64	M	G2	0	0	0	30
Sempre Viva	64	M	G4	1	0	11	30
Sempre Viva	64	F	G2	0	0	0	30
Sempre Viva	64	F	G1	0	0	0	16
Sempre Viva	64	M	G4	0	0	0	18
Sempre Viva	64	F	G2	0	0	0	16
Sempre Viva	65	M	G1	0	0	0	29
Sempre Viva	66	F	G1	0	0	0	30
Sempre Viva	66	F	G3	0	0	0	30
Sempre Viva	66	F	G1	0	0	0	30
Sempre Viva	67	F	G3	0	0	0	30
Sempre Viva	68	F	G1	0	0	0	30
Sempre Viva	68	F	G3	0	0	0	30
Sempre Viva	68	F	G3	0	0	0	30
Sempre Viva	68	M	G5	2	2	96	30
Sempre Viva	68	M	G5	0	0	0	30
Sempre Viva	69	F	G3	0	0	0	30
Sempre Viva	72	M	G3	0	0	3	30
Sempre Viva	79	F	G1	0	0	0	30

**Age (yrs)**	
**Gender**	F - female
	M - male
**G-Score (POC**	G1 -negative
	G2,G3 - trace
	G4-G10 - positive
**SmKK1**	Number of S. mansoni eggs on the first Kato-Katz slide
**SmKK12**	Number of S. mansoni eggs on the second Kato-Katz slide
**SmHTX**	Number of S. mansoni eggs on Helmintex
**g**	Amount of stool (gram) examined by Helmintex
