# Supplementary material for: Accuracy of the urine point-of-care circulating cathodic antigen assay for diagnosing Schistosomiasis mansoni infection in Brazil: A multicenter study
Source: Rev Soc Bras Med Trop. 2023 Jan 23;56:e0238-2022. doi: 10.1590/0037-8682-0238-2022 (PMC9870275; doi:10.1590/0037-8682-0238-2022)
Supplement: Supplementary file 2 [file 1678-9849-rsbmt-56-e0238-2022-supp2.pdf]

Performance parameters using HTX as reference
MedCalc Diagnostic Test Evaluation Calculator
(https://www.medcalc.org/calc/diagnostic_test.php Accessed 24 March 2022)

Bom Jesus - Cametá, PA

POC-ECOt+

	**Disease**				
**Test**	**Present**	**n**	**Absent**	**n**	**Total**
**Positive**	True Positive	a= 1	False Positive	c= 33	a + c = 34
**Negative**	False Negative	b= 0	True Negative	d= 213	b + d = 213
**Total**		a + b = 1		c + d = 246	

**Statistic**	**Value**	**95% CI**
**Sensitivity**	100.00%	2.50% to 100.00%
**Specificity**	86.59%	81.68% to 90.58%
**Positive Likelihood Ratio**	7.45	5.43 to 10.24
**Negative Likelihood Ratio**	0	
**Disease prevalence (*)**	0.40%	0.01% to 2.23%
**Positive Predictive Value (*)**	2.94%	2.16% to 4.00%
**Negative Predictive Value (*)**	100.00%	
**Accuracy (*)**	86.64%	81.75% to 90.62%

POC-ECOt-

	**Disease**				
**Test**	**Present**	**n**	**Absent**	**n**	**Total**
**Positive**	True Positive	a= 0	False Positive	c= 0	a + c = 0
**Negative**	False Negative	b= 1	True Negative	d= 246	b + d = 247
**Total**		a + b = 1		c + d = 246	

**Statistic**	**Value**	**95% CI**
**Sensitivity**	0.00%	0.00% to 97.50%
**Specificity**	100.00%	98.51% to 100.00%
**Positive Likelihood Ratio**		
**Negative Likelihood Ratio**	1	1.00 to 1.00
**Disease prevalence (*)**	0.40%	0.01% to 2.23%
**Positive Predictive Value (*)**		
**Negative Predictive Value (*)**	99.60%	99.60% to 99.60%
**Accuracy (*)**	99.60%	97.77% to 99.99%

KK

	**Disease**				
**Test**	**Present**	**n**	**Absent**	**n**	**Total**
**Positive**	True Positive	a= 1	False Positive	c= 0	a + c = 1
**Negative**	False Negative	b= 0	True Negative	d= 246	b + d = 246
**Total**		a + b = 1		c + d = 246	

**Statistic**	**Value**	**95% CI**
**Sensitivity**	100.00%	2.50% to 100.00%
**Specificity**	100.00%	98.51% to 100.00%
**Positive Likelihood Ratio**		
**Negative Likelihood Ratio**	0	
**Disease prevalence (*)**	0.40%	0.01% to 2.23%
**Positive Predictive Value (*)**	100.00%	
**Negative Predictive Value (*)**	100.00%	
**Accuracy (*)**	100.00%	98.52% to 100.00%

Palmital - Comercinho, MG

POC-ECOt+

Test	Disease Present	n	Absent	n	Total
Positive	True Positive	a= 1	False Positive	c= 1	a + c = 2
Negative	False Negative	b= 3	True Negative	d= 92	b + d = 95
Total		a + b = 4		c + d = 93	

Statistic	Value	95% CI
Sensitivity	25.00%	0.63% to 80.59%
Specificity	98.92%	94.15% to 99.97%
Positive Likelihood Ratio	23.25	1.75 to 308.33
Negative Likelihood Ratio	0.76	0.43 to 1.34
Disease prevalence (*)	4.12%	1.13% to 10.22%
Positive Predictive Value (*)	50.00%	7.01% to 92.99%
Negative Predictive Value (*)	96.84%	94.57% to 98.18%
Accuracy (*)	95.88%	89.78% to 98.87%

POC-ECOt-

Test	Disease Present	n	Absent	n	Total
Positive	True Positive	a= 1	False Positive	c= 1	a + c = 2
Negative	False Negative	b= 3	True Negative	d= 92	b + d = 95
Total		a + b = 4		c + d = 93	

Statistic	Value	95% CI
Sensitivity	25.00%	0.63% to 80.59%
Specificity	98.92%	94.15% to 99.97%
Positive Likelihood Ratio	23.25	1.75 to 308.33
Negative Likelihood Ratio	0.76	0.43 to 1.34
Disease prevalence (*)	4.12%	1.13% to 10.22%
Positive Predictive Value (*)	50.00%	7.01% to 92.99%
Negative Predictive Value (*)	96.84%	94.57% to 98.18%
Accuracy (*)	95.88%	89.78% to 98.87%

KK

Test	Disease Present	n	Absent	n	Total
Positive	True Positive	a= 1	False Positive	c= 0	a + c = 1
Negative	False Negative	b= 3	True Negative	d= 93	b + d = 96
Total		a + b = 4		c + d = 93	

Statistic	Value	95% CI
Sensitivity	25.00%	0.63% to 80.59%
Specificity	100.00%	96.11% to 100.00%
Positive Likelihood Ratio		
Negative Likelihood Ratio	0.75	0.43 to 1.32
Disease prevalence (*)	4.12%	1.13% to 10.22%
Positive Predictive Value (*)	100.00%	
Negative Predictive Value (*)	96.88%	94.63% to 98.20%
Accuracy (*)	96.91%	91.23% to 99.36%

Estreito de Miralta - Montes Claros, MG

POC-ECOt+

Test	Disease Present	n	Absent	n	Total
Positive	True Positive	a= 2	False Positive	c= 40	a + c = 42
Negative	False Negative	b= 0	True Negative	d= 56	b + d = 56
Total		a + b = 2		c + d = 96	

Statistic	Value	95% CI
Sensitivity	100.00%	15.81% to 100.00%
Specificity	58.33%	47.82% to 68.32%

**Positive Likelihood Ratio**	2.4	1.89 to 3.04
**Negative Likelihood Ratio**	0	
**Disease prevalence (*)**	2.04%	0.25% to 7.18%
**Positive Predictive Value (*)**	4.76%	3.80% to 5.96%
**Negative Predictive Value (*)**	100.00%	
**Accuracy (*)**	59.18%	48.79% to 69.01%

POC-ECOt-

	**Disease**				
**Test**	**Present**	**n**	**Absent**	**n**	**Total**
**Positive**	True Positive	a= 1	False Positive	c= 16	a + c = 17
**Negative**	False Negative	b= 1	True Negative	d= 80	b + d = 81
**Total**		a + b = 2		c + d = 96	

**Statistic**	**Value**	**95% CI**
**Sensitivity**	50.00%	1.26% to 98.74%
**Specificity**	83.33%	74.35% to 90.16%
**Positive Likelihood Ratio**	3	0.70 to 12.87
**Negative Likelihood Ratio**	0.6	0.15 to 2.41
**Disease prevalence (*)**	2.04%	0.25% to 7.18%
**Positive Predictive Value (*)**	5.88%	1.44% to 21.14%
**Negative Predictive Value (*)**	98.77%	95.23% to 99.69%
**Accuracy (*)**	82.65%	73.69% to 89.56%

KK

	**Disease**				
**Test**	**Present**	**n**	**Absent**	**n**	**Total**
**Positive**	True Positive	a= 2	False Positive	c= 0	a + c = 2
**Negative**	False Negative	b= 0	True Negative	d= 96	b + d = 96
**Total**		a + b = 2		c + d = 96	

**Statistic**	**Value**	**95% CI**
**Sensitivity**	100.00%	15.81% to 100.00%
**Specificity**	100.00%	96.23% to 100.00%
**Positive Likelihood Ratio**		
**Negative Likelihood Ratio**	0	
**Disease prevalence (*)**	2.04%	0.25% to 7.18%
**Positive Predictive Value (*)**	100.00%	
**Negative Predictive Value (*)**	100.00%	
**Accuracy (*)**	100.00%	96.31% to 100.00%

Gavião - Maranguape, CE

POC-ECOt+

	**Disease**				
**Test**	**Present**	**n**	**Absent**	**n**	**Total**
**Positive**	True Positive	a= 20	False Positive	c= 178	a + c = 198
**Negative**	False Negative	b= 2	True Negative	d= 86	b + d = 88
**Total**		a + b = 22		c + d = 264	

**Statistic**	**Value**	**95% CI**
**Sensitivity**	90.91%	70.84% to 98.88%
**Specificity**	32.58%	26.96% to 38.59%
**Positive Likelihood Ratio**	1.35	1.15 to 1.58
**Negative Likelihood Ratio**	0.28	0.07 to 1.06
**Disease prevalence (*)**	7.69%	4.88% to 11.41%
**Positive Predictive Value (*)**	10.10%	8.77% to 11.61%
**Negative Predictive Value (*)**	97.73%	91.90% to 99.39%
Accuracy (*)	37.06%	31.45% to 42.95%

POC-ECOt-

	Disease				
**Test**	**Present**	**n**	**Absent**	**n**	**Total**
**Positive**	True Positive	a= 10	False Positive	c= 63	a + c = 73
**Negative**	False Negative	b= 12	True Negative	d= 201	b + d = 213
**Total**		a + b = 22		c + d = 264	

**Statistic**	**Value**	**95% CI**
**Sensitivity**	45.45%	24.39% to 67.79%
**Specificity**	76.14%	70.53% to 81.15%
**Positive Likelihood Ratio**	1.9	1.15 to 3.16
**Negative Likelihood Ratio**	0.72	0.49 to 1.06
**Disease prevalence (*)**	7.69%	4.88% to 11.41%
**Positive Predictive Value (*)**	13.70%	8.73% to 20.84%
**Negative Predictive Value (*)**	94.37%	91.92% to 96.11%
**Accuracy (*)**	73.78%	68.27% to 78.78%

KK

	Disease				
**Test**	**Present**	**n**	**Absent**	**n**	**Total**
**Positive**	True Positive	a= 6	False Positive	c= 1	a + c = 7
**Negative**	False Negative	b= 16	True Negative	d= 263	b + d = 279
**Total**		a + b = 22		c + d = 264	

**Statistic**	**Value**	**95% CI**
**Sensitivity**	27.27%	10.73% to 50.22%
**Specificity**	99.62%	97.91% to 99.99%
**Positive Likelihood Ratio**	72	9.07 to 571.66
**Negative Likelihood Ratio**	0.73	0.57 to 0.94
**Disease prevalence (*)**	7.69%	4.88% to 11.41%
**Positive Predictive Value (*)**	85.71%	43.04% to 97.94%
**Negative Predictive Value (*)**	94.27%	92.71% to 95.50%
**Accuracy (*)**	94.06%	90.65% to 96.50%

Itaquara - Itaquara, BA

POC-ECOt+

	Disease				
**Test**	**Present**	**n**	**Absent**	**n**	**Total**
**Positive**	True Positive	a= 38	False Positive	c= 88	a + c = 126
**Negative**	False Negative	b= 8	True Negative	d= 155	b + d = 163
**Total**		a + b = 46		c + d = 243	

**Statistic**	**Value**	**95% CI**
**Sensitivity**	82.61%	68.58% to 92.18%
**Specificity**	63.79%	57.40% to 69.83%
**Positive Likelihood Ratio**	2.28	1.84 to 2.82
**Negative Likelihood Ratio**	0.27	0.14 to 0.52
**Disease prevalence (*)**	15.92%	11.89% to 20.65%
**Positive Predictive Value (*)**	30.16%	25.87% to 34.83%
**Negative Predictive Value (*)**	95.09%	91.11% to 97.34%
**Accuracy (*)**	66.78%	61.03% to 72.19%

POC-ECOt-

	Disease				
**Test**	**Present**	**n**	**Absent**	**n**	**Total**
**Positive**	True Positive	a= 12	False Positive	24	a + c = 36
**Negative**	False Negative	b= 34	True Negative	d= 219	b + d = 253
**Total**		a + b = 46		c + d = 243	

**Statistic**	**Value**	**95% CI**
**Sensitivity**	26.09%	14.27% to 41.13%

**Specificity**	90.12%	85.66% to 93.57%
**Positive Likelihood Ratio**	2.64	1.42 to 4.90
**Negative Likelihood Ratio**	0.82	0.69 to 0.98
**Disease prevalence (*)**	15.92%	11.89% to 20.65%
**Positive Predictive Value (*)**	33.33%	21.24% to 48.10%
**Negative Predictive Value (*)**	86.56%	84.37% to 88.49%
**Accuracy (*)**	79.93%	74.84% to 84.39%

KK

	**Disease**				
**Test**	Present	n	Absent	n	Total
**Positive**	True Positive	a= 13	False Positive	c= 5	a + c = 18
**Negative**	False Negative	b= 33	True Negative	d= 238	b + d = 271
Total		a + b = 46		c + d = 243	

**Statistic**	**Value**	**95% CI**
**Sensitivity**	28.26%	15.99% to 43.46%
**Specificity**	97.94%	95.26% to 99.33%
**Positive Likelihood Ratio**	13.73	5.14 to 36.67
**Negative Likelihood Ratio**	0.73	0.61 to 0.88
**Disease prevalence (*)**	15.92%	11.89% to 20.65%
**Positive Predictive Value (*)**	72.22%	49.34% to 87.41%
**Negative Predictive Value (*)**	87.82%	85.74% to 89.64%
**Accuracy (*)**	86.85%	82.40% to 90.52%

Cajueirinho - Indiaroba, SE

POC-ECOt+

	**Disease**				
**Test**	**Present**	**n**	**Absent**	**n**	**Total**
**Positive**	True Positive	a= 55	False Positive	c= 99	a + c = 154
**Negative**	False Negative	b= 24	True Negative	d= 77	b + d = 101
**Total**		a + b = 79		c + d = 176	

**Statistic**	**Value**	**95% CI**
**Sensitivity**	69.62%	58.25% to 79.47%
**Specificity**	43.75%	36.30% to 51.42%
**Positive Likelihood Ratio**	1.24	1.02 to 1.50
**Negative Likelihood Ratio**	0.69	0.48 to 1.01
**Disease prevalence (*)**	30.98%	25.36% to 37.05%
**Positive Predictive Value (*)**	35.71%	31.36% to 40.32%
**Negative Predictive Value (*)**	76.24%	68.83% to 82.34%
Accuracy (*)	51.76%	45.45% to 58.04%

POC-ECOt-

	**Disease**				
**Test**	**Present**	**n**	**Absent**	**n**	**Total**
**Positive**	True Positive	a= 44	False Positive	c= 55	a + c = 99
**Negative**	False Negative	b= 35	True Negative	d= 121	b + d = 156
**Total**		a + b = 79		c + d = 176	

**Statistic**	**Value**	**95% CI**
**Sensitivity**	55.70%	44.08% to 66.88%
**Specificity**	68.75%	61.34% to 75.51%
**Positive Likelihood Ratio**	1.78	1.33 to 2.39
**Negative Likelihood Ratio**	0.64	0.49 to 0.84
**Disease prevalence (*)**	30.98%	25.36% to 37.05%
**Positive Predictive Value (*)**	44.44%	37.34% to 51.78%
**Negative Predictive Value (*)**	77.56%	72.59% to 81.86%
**Accuracy (*)**	64.71%	58.50% to 70.57%

KK

	Disease				
**Test**	**Present**	**n**	**Absent**	**n**	**Total**
**Positive**	True Positive	a= 29	False Positive	c= 2	a + c = 31
**Negative**	False Negative	b= 50	True Negative	d= 174	b + d = 224
**Total**		a + b = 79		c + d = 176	

**Statistic**	**Value**	**95% CI**
**Sensitivity**	36.71%	26.14% to 48.31%
**Specificity**	98.86%	95.96% to 99.86%
**Positive Likelihood Ratio**	32.3	7.90 to 132.07
**Negative Likelihood Ratio**	0.64	0.54 to 0.76
**Disease prevalence (*)**	30.98%	25.36% to 37.05%
**Positive Predictive Value (*)**	93.55%	78.01% to 98.34%
**Negative Predictive Value (*)**	77.68%	74.62% to 80.47%
**Accuracy (*)**	79.61%	74.13% to 84.38%

Jaguaritira - Malacacheta, MG

POC-ECOt+

	Disease				
**Test**	**Present**	**n**	**Absent**	**n**	**Total**
**Positive**	True Positive	a= 113	False Positive	c= 74	a + c = 187
**Negative**	False Negative	b= 22	True Negative	d= 33	b + d = 55
**Total**		a + b = 135		c + d = 107	

**Statistic**	**Value**	**95% CI**
**Sensitivity**	83.70%	76.37% to 89.50%
**Specificity**	30.84%	22.27% to 40.50%
**Positive Likelihood Ratio**	1.21	1.05 to 1.40
**Negative Likelihood Ratio**	0.53	0.33 to 0.85
**Disease prevalence (*)**	55.79%	49.28% to 62.14%
**Positive Predictive Value (*)**	60.43%	56.87% to 63.88%
**Negative Predictive Value (*)**	60.00%	48.23% to 70.71%
**Accuracy (*)**	60.33%	53.86% to 66.54%

POC-ECOt-

	Disease				
**Test**	**Present**	**n**	**Absent**	**n**	**Total**
**Positive**	True Positive	a= 92	False Positive	c= 47	a + c = 139
**Negative**	False Negative	b= 43	True Negative	d= 60	b + d = 103
**Total**		a + b = 135		c + d = 107	

**Statistic**	**Value**	**95% CI**
**Sensitivity**	68.15%	59.58% to 75.90%
**Specificity**	56.07%	46.15% to 65.66%
**Positive Likelihood Ratio**	1.55	1.22 to 1.98
**Negative Likelihood Ratio**	0.57	0.42 to 0.77
**Disease prevalence (*)**	55.79%	49.28% to 62.14%
**Positive Predictive Value (*)**	66.19%	60.55% to 71.40%
**Negative Predictive Value (*)**	58.25%	50.87% to 65.28%
**Accuracy (*)**	62.81%	56.39% to 68.92%

KK

	Disease				
**Test**	**Present**	**n**	**Absent**	**n**	**Total**
**Positive**	True Positive	a= 43	False Positive	c= 6	a + c = 49
**Negative**	False Negative	b= 92	True Negative	d= 101	b + d = 193
**Total**		a + b = 135		c + d = 107	

**Statistic**	**Value**	**95% CI**

**Sensitivity**	31.85%	24.10% to 40.42%
**Specificity**	94.39%	88.19% to 97.91%
**Positive Likelihood Ratio**	5.68	2.51 to 12.84
**Negative Likelihood Ratio**	0.72	0.64 to 0.82
**Disease prevalence (*)**	55.79%	49.28% to 62.14%
**Positive Predictive Value (*)**	87.76%	76.02% to 94.19%
**Negative Predictive Value (*)**	52.33%	49.23% to 55.42%
**Accuracy (*)**	59.50%	53.03% to 65.74%

Sempre Viva - Conde, BA

POC-ECOt+

**Test**	**Disease Present**	**n**	**Absent**	**n**	**Total**
**Positive**	True Positive	a= 110	False Positive	c= 149	a + c = 259
**Negative**	False Negative	b= 25	True Negative	d= 97	b + d = 122
**Total**		a + b = 135		c + d = 246	

**Statistic**	**Value**	**95% CI**
**Sensitivity**	81.48%	73.89% to 87.64%
**Specificity**	39.43%	33.28% to 45.84%
**Positive Likelihood Ratio**	1.35	1.18 to 1.53
**Negative Likelihood Ratio**	0.47	0.32 to 0.69
**Disease prevalence (*)**	35.43%	30.63% to 40.47%
**Positive Predictive Value (*)**	42.47%	39.35% to 45.65%
**Negative Predictive Value (*)**	79.51%	72.50% to 85.10%
**Accuracy (*)**	54.33%	49.18% to 59.41%

POC-ECOt-

**Test**	**Disease Present**	**n**	**Absent**	**n**	**Total**
**Positive**	True Positive	a= 85	False Positive	c= 80	a + c = 165
**Negative**	False Negative	b= 50	True Negative	d= 166	b + d = 216
**Total**		a + b = 135		c + d = 246	

**Statistic**	**Value**	**95% CI**
**Sensitivity**	62.96%	54.23% to 71.11%
**Specificity**	67.48%	61.24% to 73.29%
**Positive Likelihood Ratio**	1.94	1.55 to 2.42
**Negative Likelihood Ratio**	0.55	0.43 to 0.70
**Disease prevalence (*)**	35.43%	30.63% to 40.47%
**Positive Predictive Value (*)**	51.52%	45.98% to 57.01%
**Negative Predictive Value (*)**	76.85%	72.38% to 80.79%
**Accuracy (*)**	65.88%	60.88% to 70.63%

KK

**Test**	**Disease Present**	**n**	**Absent**	**n**	**Total**
**Positive**	True Positive	a= 80	False Positive	c= 24	a + c = 104
**Negative**	False Negative	b= 55	True Negative	d= 222	b + d = 277
**Total**		a + b = 135		c + d = 246	

**Statistic**	**Value**	**95% CI**
**Sensitivity**	59.26%	50.47% to 67.63%
**Specificity**	90.24%	85.83% to 93.65%
**Positive Likelihood Ratio**	6.07	4.05 to 9.11
**Negative Likelihood Ratio**	0.45	0.37 to 0.56
**Disease prevalence (*)**	35.43%	30.63% to 40.47%
**Positive Predictive Value (*)**	76.92%	68.98% to 83.33%
**Negative Predictive Value (*)**	80.14%	76.63% to 83.24%
**Accuracy (*)**	79.27%	74.84% to 83.23%

All sites

POC-ECOt+

	Disease				
**Test**	**Present**	**n**	**Absent**	**n**	**Total**
**Positive**	True Positive	a= 340	False Positive	c= 662	a + c = 1002
**Negative**	False Negative	b=84	True Negative	d= 809	b + d = 893
**Total**		a + b = 424		c + d = 1471	

**Statistic**	**Value**	**95% CI**
**Sensitivity**	80.19%	76.07% to 83.88%
**Specificity**	55.00%	52.41% to 57.56%
**Positive Likelihood Ratio**	1.78	1.66 to 1.92
**Negative Likelihood Ratio**	0.36	0.30 to 0.44
**Disease prevalence (*)**	22.37%	20.52% to 24.32%
**Positive Predictive Value (*)**	33.93%	32.30% to 35.60%
**Negative Predictive Value (*)**	90.59%	88.78% to 92.14%
**Accuracy (*)**	60.63%	58.39% to 62.84%

POC-ECOt-

	Disease				
**Test**	**Present**	**n**	**Absent**	**n**	**Total**
**Positive**	True Positive	a= 245	False Positive	c= 286	a + c = 531
**Negative**	False Negative	b= 179	True Negative	d= 1185	b + d = 1364
**Total**		a + b = 424		c + d = 1471	

**Statistic**	**Value**	**95% CI**
**Sensitivity**	57.78%	52.92% to 62.53%
**Specificity**	80.56%	78.44% to 82.55%
**Positive Likelihood Ratio**	2.97	2.60 to 3.39
**Negative Likelihood Ratio**	0.52	0.47 to 0.59
**Disease prevalence (*)**	22.37%	20.52% to 24.32%
**Positive Predictive Value (*)**	46.14%	42.88% to 49.43%
**Negative Predictive Value (*)**	86.88%	85.52% to 88.12%
**Accuracy (*)**	75.46%	73.46% to 77.38%

KK

	Disease				
**Test**	**Present**	**n**	**Absent**	**n**	**Total**
**Positive**	True Positive	a= 175	False Positive	c= 38	a + c = 213
**Negative**	False Negative	b= 249	True Negative	d= 1433	b + d = 1682
**Total**		a + b = 424		c + d = 1471	

**Statistic**	**Value**	**95% CI**
**Sensitivity**	41.27%	36.54% to 46.13%
**Specificity**	97.42%	96.47% to 98.17%
**Positive Likelihood Ratio**	15.98	11.44 to 22.31
**Negative Likelihood Ratio**	0.6	0.56 to 0.65
**Disease prevalence (*)**	22.37%	20.52% to 24.32%
**Positive Predictive Value (*)**	82.16%	76.74% to 86.54%
**Negative Predictive Value (*)**	85.20%	84.16% to 86.18%
**Accuracy (*)**	84.85%	83.16% to 86.44%

(*) These values are dependent on disease prevalence.

Definitions

- *Sensitivity*: probability that a test result will be positive when the disease is present (true positive rate).
  = a / (a+b)
- *Specificity*: probability that a test result will be negative when the disease is not present (true negative rate).
  = d / (c+d)
- *Positive likelihood ratio*: ratio between the probability of a positive test result given the *presence* of the disease and the probability of a positive test result given the *absence* of the

- [illegible] disease, i.e.

  = True positive rate / False positive rate = Sensitivity / (1-Specificity)

- *Negative likelihood ratio*: ratio between the probability of a negative test result given the *presence* of the disease and the probability of a negative test result given the *absence* of the disease, i.e.

  = False negative rate / True negative rate = (1-Sensitivity) / Specificity

- *Positive predictive value*: probability that the disease is present when the test is positive.

$$PPV = \frac{sensitivity \times prevalence}{sensitivity \times prevalence + (1 - specificity) \times (1 - prevalence)}$$

- *Negative predictive value*: probability that the disease is not present when the test is negative.

$$NPV = \frac{specificity \times (1 - prevalence)}{(1 - sensitivity) \times prevalence + specificity \times (1 - prevalence)}$$

- *Accuracy*: overall probability that a patient is correctly classified.

  = Sensitivity × Prevalence + Specificity × (1 − Prevalence)

Sensitivity, specificity, disease prevalence, positive and negative predictive value as well as accuracy are expressed as percentages.
